# Wnt and Treg-Associated Signaling Coordinate Mucosal Regeneration and MALT Formation in a Mouse Model of Chronic Colitis

**DOI:** 10.3390/ijms27020779

**Published:** 2026-01-13

**Authors:** Nanami Watanabe, Mio Kobayashi, Tatsu Kuriki, Yuri Ebizuka, Mai Hirata, Rintaro Mizuguchi, Mio Takimoto, Bai Yidan, Mengyuan Luo, Mai Todoroki, Ma Suzanneth G. Lola, Xinyu Zou, Sha Jiang, Tetsuhito Kigata, Makoto Shibutani, Toshinori Yoshida, Tsutomu Omatsu

**Affiliations:** 1 Laboratory of Veterinary Pathology, Cooperative Department of Veterinary Medicine, Tokyo University of Agriculture and Technology, 3-5-8 Saiwai-cho, Fuchu-shi 183-8509, Tokyo, Japan; 2 Department of Basic Pathology, Fukushima Medical University, School of Medicine, 1 Hikarigaoka, Fukushima-shi 960-1295, Fukushima, Japan; 3 Center for Infectious Disease Epidemiology and Prevention Research, Faculty of Agriculture, Tokyo University of Agriculture and Technology, 3-5-8 Saiwai-cho, Fuchu-shi 183-8509, Tokyo, Japantomatsu@cc.tuat.ac.jp (T.O.); 4 Cooperative Division of Veterinary Sciences, Tokyo University of Agriculture and Technology, 3-5-8 Saiwai-cho, Fuchu-shi 183-8509, Tokyo, Japan; 5 Department of Veterinary Paraclinical Sciences, College of Veterinary Medicine, University of the Philippines, Los Banos, Laguna 4031, Philippines; 6 Joint International Research Laboratory of Animal Health and Animal Food Safety, College of Veterinary Medicine, Southwest University, 2nd Tiansheng Road, Beibei District, Chongqing 400715, China; 7 Laboratory of Veterinary Anatomy, Cooperative Department of Veterinary Medicine, Tokyo University of Agriculture and Technology, 3-5-8 Saiwai-cho, Fuchu-shi 183-8509, Tokyo, Japan; kigatat@go.tuat.ac.jp

**Keywords:** colitis, lymphoid follicle, *Lgr5*, mucosal regeneration, Treg

## Abstract

Chronic ulcerative colitis disrupts mucosal-acquired immunity; however, the relationship between mucosal regeneration and mucosa-associated lymph tissue (MALT) development remains unclear. We explored crypt responses, MALT phenotypes, and regulatory T cells (Tregs) in a mouse model of chronic colitis following two cycles of dextran sodium sulfate (DSS) exposure. The mucosal regeneration score correlated with crypt expression of Ki-67 and LGR5, submucosal FOXP3-positive Treg expression, and MALT scores. MALT can be categorized into solitary-isolated lymphoid structures, tertiary lymphoid structures, and colonic patches. Regenerative crypts adjacent to tertiary lymphoid structures exhibit reduced expression of Ki-67, LGR5, and SOX9, which might favor mucosal differentiation. These findings were further supported by correlations between crypt stem cell- and Treg-related colonic gene expression of *Lgr5*, *Sox9*, *Wnt6*, *Ccl20*, and *IL10*, and between *Tgfb1* and *Cxcl13*. These results suggested that chronic colitis is repaired by stem cell-mediated mucosal regeneration and differentiation, potentially driven by the development of MALT-containing Tregs.

## 1. Introduction

Inflammatory bowel disease (IBD) is a chronic inflammatory condition of the gastrointestinal tract that is clinically categorized into Crohn’s disease (CD) and ulcerative colitis (UC) [1]. Patients with IBD commonly experience recurrent abdominal pain, diarrhea, bloody stools, weight loss, and a reduced quality of life. IBD incidence and prevalence have increased significantly in the latter half of the 20th century, and it is recognized as one of the most common gastrointestinal disorders. Its incidence has increased in newly industrialized countries since the early 21st century [2]. UC primarily affects students and working adults, imposing substantial social and economic burdens. Despite its high prevalence, effective treatments for UC remain limited. The pathogenesis of UC is attributed to the complex interplay of endogenous and exogenous factors, including genetic predisposition, environmental influences, lifestyle involvement, gut microbiota dysregulation, and immune system abnormalities [3,4,5]. However, the precise mechanisms underlying the development of the disease remain not fully understood.

Several murine models of colitis, including dextran sodium sulfate (DSS) treatment, have been developed to elucidate the mechanisms underlying UC pathogenesis and evaluate potential therapeutic interventions [6,7]. The murine DSS model of colitis is considered superior owing to its rapid induction, simplicity, reproducibility, and resemblance to the pathology of UC [8]. The severity and stage can be adjusted by varying the concentration and duration of DSS administration, thereby enabling the induction of acute, chronic, and recurrent colitis [9]. Acute colitis develops when a relatively high dose of DSS is administered to mice for 6–10 days, whereas chronic colitis requires repeated cycles of low-to-medium doses of DSS for approximately 1 week, followed by recovery periods with sterile water for 1 or 2 weeks [8]. Chronic DSS-induced colitis is characterized by mucus discharge, reduced goblet cells, distortion and atrophy of crypts, chronic inflammatory infiltrates, and lymphoid aggregates [7]. The lymphoid aggregates mature into mucosa-associated lymphoid tissues (MALTs), including secondary and tertiary lymphoid tissues [10,11,12], which might be useful for determining acquired immunity in chronic colitis.

Regulatory T cells (Tregs) play a role in maintaining immune tolerance and homeostasis, thereby reducing inflammation in the intestinal mucosa [4,13,14]. Tregs are characterized by expression of the transcriptional regulator forkhead box P3 (FOXP3), which regulates both innate and acquired immunity by modulating multiple immune cells, including neutrophils, monocytes, antigen-presenting cells, B cells, and T cells [15]. Accumulating evidence indicates that Tregs protect the colonic mucosa from harmful microorganisms and maintain intestinal homeostasis, whereas dysregulation of Tregs may progress to IBD [16]. While Treg numbers are increased in the inflamed mucosa of patients with IBD, they are significantly decreased in other inflammatory diseases, such as diverticulitis [16]. Effective therapeutic approaches to treat IBD, such as anti-tumor necrosis factor therapy, have been shown to increase Treg populations in the colonic mucosa of patients with IBD [17]. However, the relationship among mucosal regeneration, Tregs, and MALT in murine models of chronic colitis remains poorly understood.

In the present study, we explored the relationship between mucosal regeneration, repair of erosions and ulcers, and MALT development in a mouse model of DSS-induced colitis. Mucosal regeneration was evaluated using markers of cell proliferation and stem cell activity with Ki-67, leucine-rich repeat-containing G-protein coupled receptor 5 (LGR5), and sex-determining region Y box 9 (SOX9) in the crypts. LGR5, a target gene of the Wnt signaling pathway, is expressed in crypt base columnar (CBC) cells, which generate all differentiated intestinal cell types [18,19,20,21]. SOX9, another transcription factor regulated by Wnt signaling, is expressed in crypt base cells [22,23,24]. Both LGR5- and SOX9-expressed CBC cells regulate crypt stemness [19,20,21]. We histologically categorized MALT into three types—isolated lymphoid follicles (ILFs), colonic patch (CP), and tertiary lymphoid tissue (tLT)—according to established criteria [11,25,26] and compared those with LGR5- and SOX9-expressing CBC and the gene expression levels of MALT-related cytokines including *Cxcl13* and *Ccl20* [27]. Furthermore, we quantitatively assessed FOXP3-positive Tregs and their target genes, including *Tgfb1* and *Il10*, to better understand immunosuppressive roles in mucosal regeneration. We analyzed gene expression in the Wnt/β-catenin signaling pathway, including epithelial cell-associated genes (*Wnt3/6/9b*) and stromal cell-associated genes (*Wnt2b/4/5a/5b*), as well as the Wnt agonist R-spongin (Rspos) genes (*Rspo2/3*), which are essential for LGR5+ stem cell maintenance [21,28,29]. This study aimed to provide a comprehensive understanding of the interplay between mucosal regeneration and MALT and to identify potential therapeutic targets.

## 2. Results

### 2.1. DSS-Induced Chronic Colitis Causes Periodic Diarrhea and Bloody Stools

First, our experiments confirmed the clinical signs in the murine model of chronic UC. The results of Experiment I (pathological examination) showed reparative changes in DAI, as indicated by scores for body weight loss, diarrhea, and fecal blood. The fecal blood score and DAI were evident compared to the control (Supplemental Figure S1A–E and Tables S1–S4), even though there were no changes in the survival rate (Supplemental Figure S1F). One control presented high scores during the study due to spontaneous causes; these might be a limitation when evaluating DAI in the treated groups. Although there were no significant changes in colon length, its weight, or spleen weight, colon length was shortened in the low and high-dose groups (Supplemental Table S5 and Figure S2). In Experiment II (for gene expression analysis), similar findings were obtained for DAI, body weight, survival rate, colon length, and colon weight (Supplemental Table S6 and Figure S3A–H).

### 2.2. DSS-Induced Chronic Colitis Induces Mucosal Regeneration

Next, we explored the pathological changes in the DSS-induced colitis model. We observed mucosal loss, edema, and inflammation in a dose-dependent manner, followed by mucosal regeneration, as indicated by histopathological scores in Experiment I (Supplemental Figure S4A). Immunohistochemistry demonstrated the expression of cell adhesion, E-cadherin, and β-catenin in the intact and regenerative crypts, and interstitial infiltration of CD3-expressing T cells, FOXP3-expressing Tregs, and PAX5 and CD20-expressing B cells in the treated mice (Supplemental Figures S4B and S5). E-cadherin and β-catenin expression in regenerative mucosa was similar to that in the control. T cells, B cells, and Tregs infiltrated the lamina propria and submucosal tissues in the DSS treatment groups.

### 2.3. DSS-Induced Chronic Colitis Increases MALT

**Figure 1:**
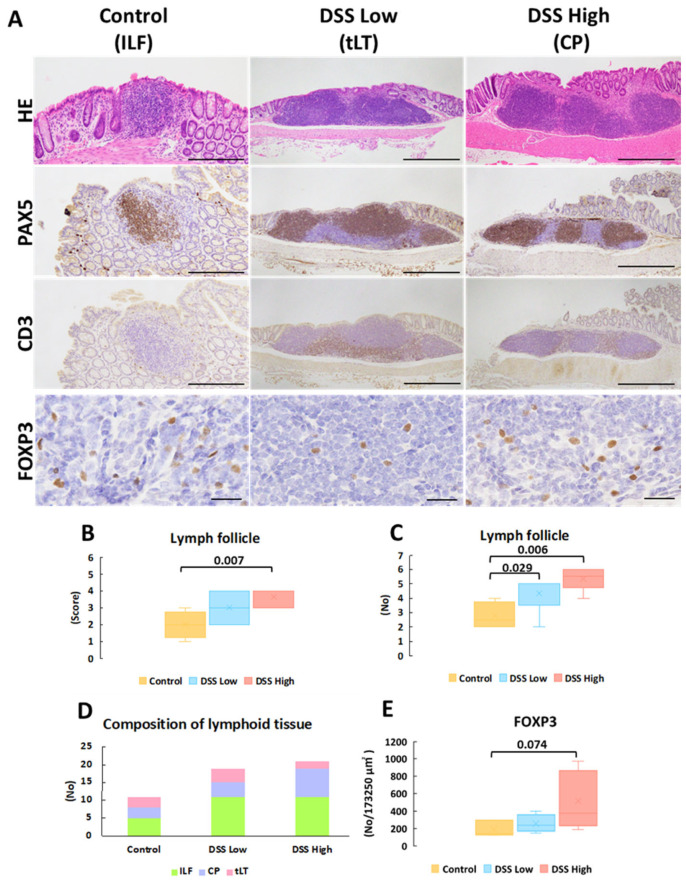
Experiment I. Representative images of histopathology and immunohistochemistry of mucosa-associated lymphoid tissues. (**A**) HE stains and immunohistochemical staining of PAX5, CD3, and FOXP3 of lymph follicles in control, DSS low, and DSS high, which represent ILF, CP, and LT, respectively. (**B**) The score of lymph follicle size (see Supplemental Figure S6 and Table S3). (**C**) The number of lymph follicles. (**D**) The composition of lymph follicles in each group. (**E**) The number of FOXP3-positive cells per 173,250 μm^2^ (165 μm × 210 μm × 5 fields) of lymph follicles. Significant differences between each group are shown as *p* values (Mann–Whitney U test with the Bonferroni correction for comparison between the three groups for the score and number of lymph follicles or Dunnet’s multiple comparison test for FOXP3-positive cells). Bar = 500 μm (HE, PAX5 and CD3 in DSS Low and DSS high), 200 μm (HE, PAX5 and CD3 in control), and 50 μm (FOXP3 in all the groups). Control, control group (*n* = 4); CP; colonic patch; DSS low, the low-dose DSS-treated group (*n* = 6); and DSS high; high-dose DSS-treated group (*n* = 6); FOXP3, forkhead box P; HE, hematoxylin and eosin; ILF; isolated lymphatic follicle; PAX5; paired box protein 5; tLT, tertiary lymphoid tissue.

When we noted colitis and regenerative crypts, we noticed that MALT was scattered in the control and DSS-treated groups in Experiment I. The score and number of MALT increased in a dose-dependent manner in the treated mice (Figure 1A–E; Supplemental Figure S6A–H). Immunohistochemistry showed the distribution of T and B cells in MALT, which were categorized into three phenotypes based on previously reported criteria [11,25,26]: ILFs were single clusters of B cells with scattered T cells within the mucosa; CP consisted of multiple B cell follicles with distinct T cell regions in the submucosa; and tLT was a large lymphoid tissue in which multiple B cell follicles in the mucosa are separated by distinct T cell regions (Figure 1A). Although no significant changes were detected in the population of each type of MALT, ILF and tLT were evident in the low-dose group, and ILF and CP were evident in the high-dose group (Figure 1D). The number of FoxP3-expressing Tregs increased in a dose-dependent manner, with no significant differences between the groups (Figure 1E).

### 2.4. DSS-Induced Chronic Colitis Enhances Mucosal Regeneration with Cell Proliferation and Stem Cell Activity

**Figure 2:**
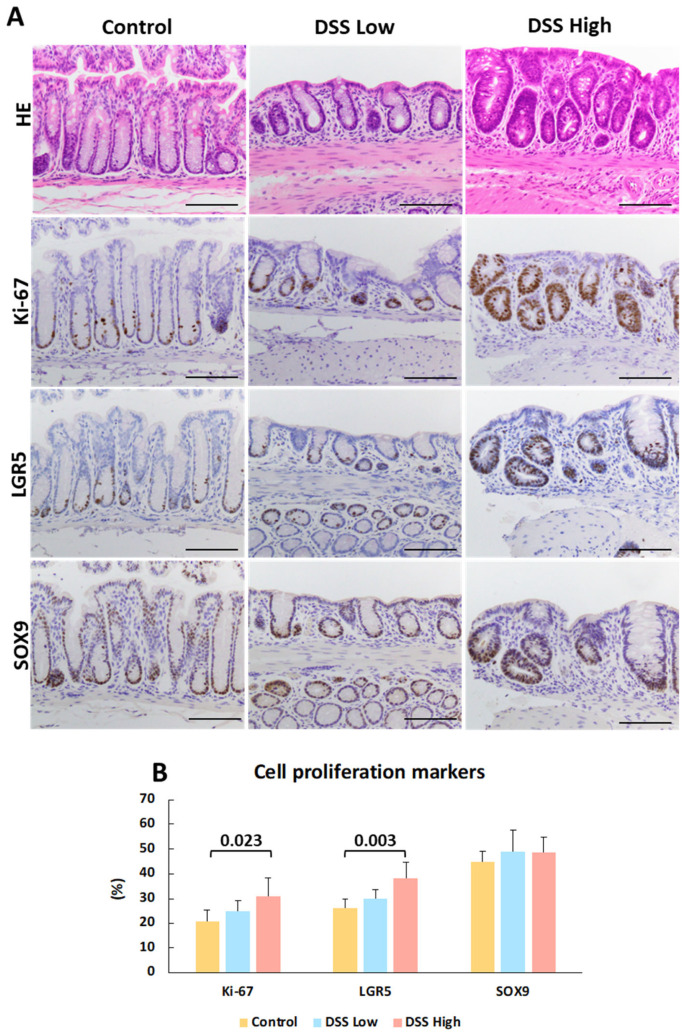
Experiment I. Representative images of histopathology and immunohistochemistry of crypts and quantitative analysis of cell proliferation and stem cell activities. (**A**) HE stains and immunohistochemical staining of Ki-67, LGR5, and SOX9 in control, DSS low, and DSS high. DSS low and DSS high represent regenerative crypts. (**B**) Percentage of Ki-67-, LGR5-, and SOX9-positive cells per 50 total cells in the crypts. The data represent the mean values and standard deviations. Significant differences between groups are shown as *p* values (Dunnett’s multiple comparison test). Bar = 100 μm. Control, control group (*n* = 4); DSS low, low-dose DSS-treated group (*n* = 6); and DSS High; high-dose DSS-treated group (*n* = 6); HE, hematoxylin and eosin; LGR5, leucine-rich repeat-containing G-protein coupled receptor 5; SOX9, sex-determining region Y box 9.

**Figure 3:**
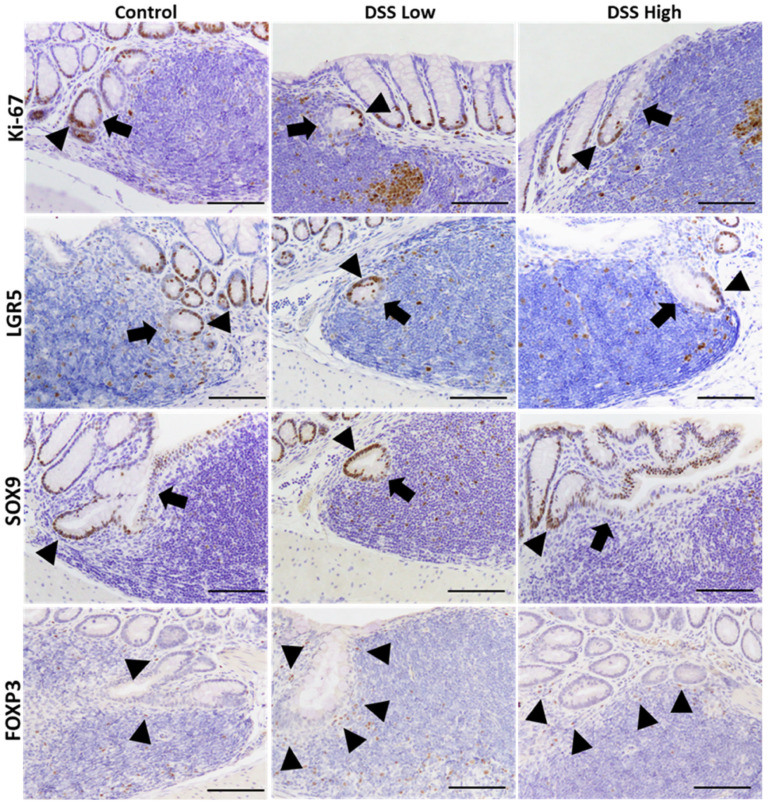
Experiment I. Representative images of immunohistochemical staining of Ki-67, LGR5, SOX9, and FOXP3 in the crypts close to tLT in the control, DSS low, and DSS high. Cells in the crypts tend to be negative for cells close to tLT and positive for cells on the opposite side. Arrowheads indicate positive epithelial cells, while arrows indicate negative epithelial cells in the crypts. Bar = 100 μm. Control, control group (*n* = 4); DSS low, low-dose DSS-treated group (*n* = 6); and DSS High; high-dose DSS-treated group (*n* = 6); FOXP3, forkhead box P; HE, hematoxylin and eosin; LGR5, leucine-rich repeat-containing G-protein coupled receptor 5; SOX9, sex-determining region Y box 9.

Immunohistochemistry was conducted for Ki-67, LGR5, and SOX9 to elucidate cell proliferation and stem cell marker expression in regenerative crypts in Experiment I (Figure 2A). Positive signals were observed in the crypt bases of control mice and in the regenerative crypts of treated mice. Ki-67 and LGR5 expression increased in a dose-dependent manner, whereas SOX9 expression was consistently observed in the control and treated groups (Figure 2B). The most critical finding of immunohistochemical staining for Ki-67, LGR5, and SOX9 were that the number of positive cells decreased in the crypts adjacent to the tLT compared with the number of positive cells outside the tLT. Regenerative crypts were also found to partially encroach upon the tLT (Figure 3; Supplemental Figure S7).

### 2.5. Correlation Analysis of Mucosal Regeneration and Markers for Stem Cell and Treg

**Figure 4:**
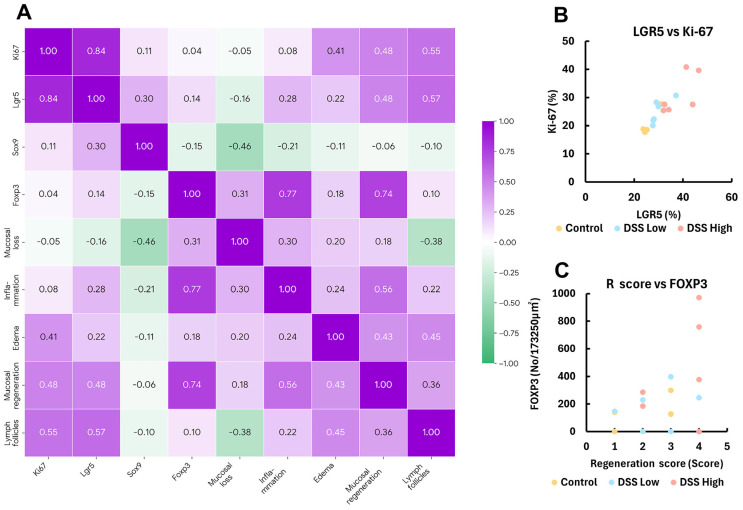
Experiment I. Correlation plots of the respective positive cell rates for LGR5, SOX9, Ki-67, and FOXP3, histopathology score, and lymph follicle score in control, DSS low, and DSS high. (**A**) The heatmap with Spearman’s rank correlation coefficient. (**B**,**C**) Correlation plots of Ki-67 and LGR5-positive crypt cells, and FOXP3-positive Treg and regenerative score. Control, control group (*n* = 4); DSS low, low-dose DSS-treated group (*n* = 6); and DSS High; high-dose DSS-treated group (*n* = 6); FOXP3, forkhead box P; LGR5, leucine-rich repeat-containing G-protein coupled receptor 5; R score, Regeneration score; SOX9, sex-determining region Y box 9.

Given that mucosal regeneration and MALT development are associated with chronic colitis, we examined correlations among several histopathological and immunohistochemical parameters in Experiment I. The association between positive cell rates of Ki-67 and LGR5 was determined by the Spearman’s correlation coefficients, which were very strong for each parameter (correlation coefficient *r*_s_ = 0.84, *p* < 0.01) (Figure 4A,B; Supplemental Table S7 and Figure S8). Both markers were positively correlated with the mucosal regeneration score; however, no significant differences were observed (*r*_s_ = 0.48, *p* = 0.06 for Ki67 and *r*_s_ = 0.48, *p* = 0.06 for LGR5).

There was also a strong correlation between the number of FOXP3-positive cells in MALT and mucosal regeneration score (*r*_s_ = 0.74, *p* < 0.01) (Figure 4A,C; Supplemental Table S7 and Figure S8). The association between the lymph follicle score and positive cell rates of Ki-67 and LGR5 was determined by correlation coefficients, which also demonstrated a strong correlation (*r*_s_ = 0.55, *p* < 0.05 for Ki-67, and *r*_s_ = 0.57, *p* < 0.05 for Lgr5) (Figure 4A; Supplemental Table S7 and Figure S8).

### 2.6. Gene Expression Analysis and Correlation of Each Parameter

**Figure 5:**
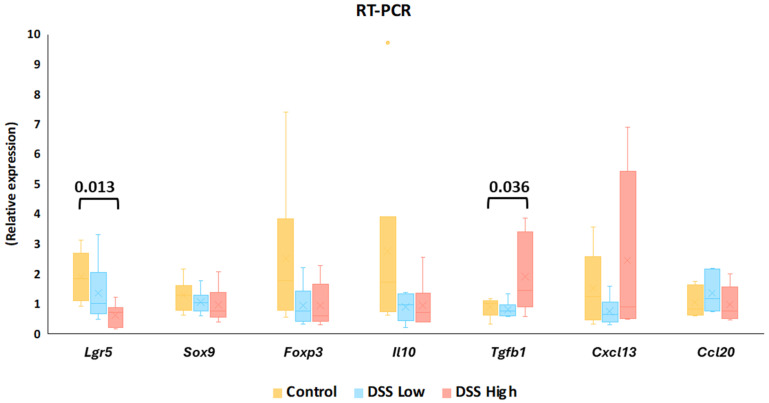
Experiment II. Gene expression levels of *Lgr5*, *Sox9*, *Foxp3*, *Il10*, *Tgfb1*, *Cxcl13*, and *Ccl20* in control, DSS low, and DSS high by reverse transcription polymerase chain reaction (RT-PCR). The data represent box whisker plot with the interquartile range, maximum, minimum, and yellow dots, outliers). Significant differences between groups are shown as *p* values (Dunnett’s multiple comparison test). Control, control group (*n* = 6); *Ccl20*, chemokine (C-C motif) ligand 20, *Cxcl13*, chemokine (C-X-C motif) ligand 13; DSS low, low-dose DSS-treated group (*n* = 6); and DSS high; high-dose DSS-treated group (*n* = 6); *Foxp3*, forkhead box P; *Il-10*, interleukin 10; *Lgr5*, leucine-rich repeat-containing G-protein coupled receptor 5; *Sox9*, sex-determining region Y box 9; *Tgfb1*, transforming growth factor beta 1.

Using mucosal samples from the chronic colitis model in Experiment II, we next investigated gene expression in stem and progenitor cells, Wnt/β-catenin signaling, and Treg-related cytokines. RT-PCR showed that *Lgr5* and *Tgfb1* expression was significantly decreased and increased, respectively, in the high-dose group compared to the control group (Figure 5). However, there were no significant differences in other genes between the groups (Figure 5; Supplemental Tables S8 and S9).

To further explore the involvement of gene expression in mucosal regeneration and MALT formation, we focused on the expression of stem cell/cell differentiation-, Wnt signaling-, Treg regulator-, and lymphoid-related genes and assessed their correlations with other gene expression (Figure 6A; Supplemental Table S10 and Figures S9–S16).

**Figure 6:**
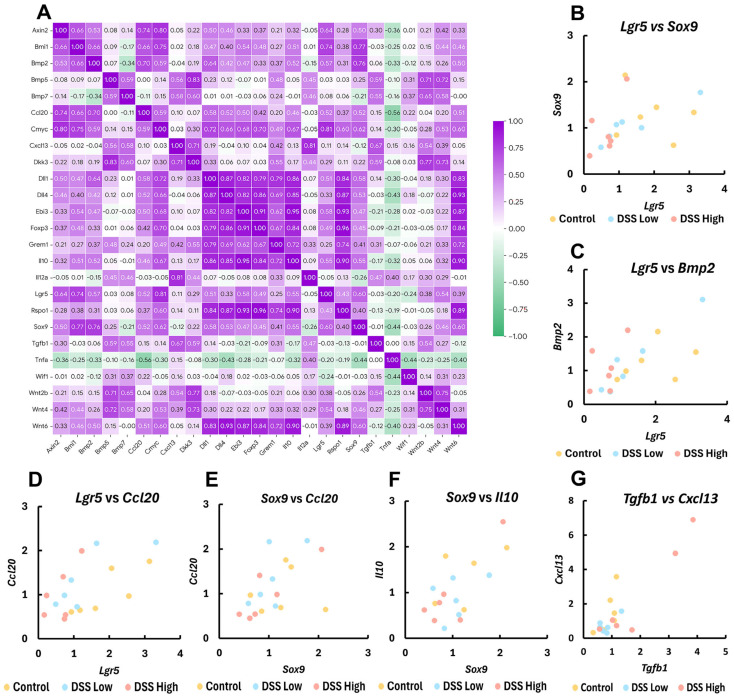
Experiment II. Correlation plots of gene expression levels by RT-PCR for crypt stem cell-, cell differentiation-, Treg regulators, and lymphoid-related genes. (**A**) The heatmap with Spearman’s rank correlation coefficient; *Axin2*, axis inhibition protein 2; *Bmi1*, BMI1 proto-oncogene; *Bmp5*, bone morphogenetic protein 5; *Bmp7*, bone morphogenetic protein 7; *Cmyc*, MYC proto-oncogene; *Dkk3*, dickkopf WNT signaling pathway inhibitor 3; *Dll1*, delta like canonical Notch ligand 1; *Dll4*, delta like canonical Notch ligand 4; *Ebi3*, Epstein-Barr virus induced 3; *Grem1*, gremlin 1; *Il12a*, interleukin 12A; *Rspo1*, R-spondin 1; *Tnfa*, tumor necrosis factor alpha; *Wif1*, WNT inhibitory factor 1; *Wnt2b*, Wnt family member 2B; *Wnt4*, Wnt family member 4; and *Wnt6*, Wnt family member 6. (**B**–**G**) Correlation plots of *Lgr5*, *Sox9*, *Bmp2*, *Ccl20*, *Il10*, *Tgfb1*, and *Cxcl13* in control, DSS low, and DSS high. Control, control group (*n* = 6); DSS low, the low-dose DSS-treated group (*n* = 6); and DSS high, the high-dose DSS-treated group (*n* = 6); *Ccl20*, chemokine (C-C motif) ligand 20, *Cxcl13*, chemokine (C-X-C motif) ligand 13; *Foxp3*, forkhead box P; *Il-10*, interleukin 10; *Lgr5*, leucine-rich repeat-containing G-protein coupled receptor 5; *Sox9*, sex-determining region Y box 9; *Tgfb1*, transforming growth factor beta 1.

*Lgr5* moderately correlated with *Sox9* (*r*_s_ = 0.60, *p* < 0.01) (Figure 6B). *Lgr5* and *Sox9* positively correlated with *Axin2* (*r*_s_ = 0.64, *p* < 0.01 for *Lgr5*; *r*_s_ = 0.50, *p* < 0.05 for *Sox9*), *Bmi1* (*r*_s_ = 0.74, *p* < 0.01 for *Lgr5*; *r*_s_ = 0.77, *p* < 0.01 for *Sox9*), *Bmp2* (*r*_s_ = 0.57, *p* < 0.05 for *Lgr5*; *r*_s_ = 0.76, *p* < 0.01 for *Sox9*) (Figure 6C), *cMyc* (*r*_s_ = 0.81, *p* < 0.01 for *Lgr5*; *r*_s_ = 0.62, *p* < 0.01 for *Sox9*), and *Dll1* (*r*_s_ = 0.51, *p* < 0.05 for *Lgr5*; *r*_s_ = 0.58, *p* < 0.05 for *Sox9*). *Lgr5* also positively correlated with *Wnt4* (*r*_s_ = 0.54, *p* < 0.05), and *Sox9* positively correlated with *Wnt6* (*r*_s_ = 0.60, *p* < 0.01) and *Dll4* (*r*_s_ = 0.53, *p* < 0.05). *Wnt6* positively correlated with *Rspo1* (*r*_s_ = 0.89, *p* < 0.01), *Bmp2* (*r*_s_ = 0.50, *p* < 0.05), *Grem1* (*r*_s_ = 0.72, *p* < 0.01), *cMyc* (*r*_s_ = 0.60, *p* < 0.01), *Dlli1* (*r*_s_ = 0.83, *p* < 0.01), and *Dll4* (*r*_s_ = 0.93, *p* < 0.01).

*Lgr5*, *Sox9* and *Wnt6* also positively correlated with immunomodulator genes: *Ccl20* (*r*_s_ = 0.52, *p* < 0.05 for *Lgr5*; *r*_s_ = 0.52, *p* < 0.05 for *Sox9*; *r*_s_ = 0.51, *p* < 0.05 for *Wnt6*) and *Il10* (*r*_s_ = 0.55, *p* < 0.05 for *Lgr5*; *r*_s_ = 0.55, *p* < 0.05 for *Sox9*; *r*_s_ = 0.90, *p* < 0.01 for *Wnt6*) (Figure 6D–F). *Lgr5* also positively correlated with *Foxp3* (*r*_s_ = 0.49, *p* < 0.05), and *Wnt6* also positively correlated with *Ebi3* (*r*_s_ = 0.87, *p* < 0.01) and *Foxp3* (*r*_s_ = 0.84, *p* < 0.01). *Tgfb1*, *Foxp3*, *Cxcl13*, and *Ccl20* positively correlated with *Cxcl13* (*r*_s_ = 0.67, *p* < 0.01) (Figure 6G), *Il10* (*r*_s_ = 0.84, *p* < 0.01), *Il12a* (*r*_s_ = 0.81, *p* < 0.01), and *Ebi3* (*r*_s_ = 0.50, *p* < 0.05), respectively. Significant negative correlations in gene expression were limited and were observed only for *Ccl20* and *Tnfa* (*r*_s_ = −0.56, *p* < 0.05).

Additionally, significant correlations were observed across multiple other gene-expression levels (Figure 6A; Supplemental Table S10 and Figures S9–S16).

## 3. Discussion

Patients with UC experience a chronic disease course characterized by repeated cycles of remission and relapse that gradually diminish their QOL. To replicate these pathological conditions in animal models, chronic colitis can be induced by repeated exposure to low to moderate doses of DSS, interspersed with recovery periods, which can be repeated for several cycles [6,7,8,9]. In this study, we used a two-cycle DSS model with a 5-day administration (1.5% and 3%), followed by 5 days of recovery to observe chronic colitis with mucosal healing. The main findings demonstrated an association between mucosal regeneration and the expression of the stem cell marker LGR5 in intestinal crypts, along with increased MALT and Treg populations. These findings were further supported by correlations between crypt stem cell- and Treg-related colonic gene expression of *Lgr5*, *Sox9*, *Wnt6*, *Ccl20*, and *IL10*, and between *Tgfb1* and *Cxcl13*. These data provide insight into the relationship between mucosal regeneration mechanisms and the regulation of intestinal acquired immunity in a UC model.

Research using the crypt stem cell approach has highlighted the small intestine, where the role of LGR5-expressed CBC and their supporting Paneth cells in mucosal healing is well understood [18]. Although the mechanisms governing mucosal repair in the large intestine are not fully understood, LGR5- and SOX9-expressing crypt cells may play crucial roles in maintaining colon crypt homeostasis [18,23,30]. LGR5-expressed CBC is highly sensitive to colonic stress. After crypt damage induced by a 5-day exposure to DSS, LGR5+ stem cells were initially depleted, but reappeared on day 5 and reached normal levels by day 6 of the recovery period [31]. Similar patterns have been reported in both acute and chronic DSS-induced colitis models [32]. Our data demonstrated that mucosal regeneration is closely correlated with the number of LGR5-expressed epithelial cells with a positive correlation of *Lgr5* and *Sox9*, following the chronic phase of DSS-mediated colitis. In the small intestine, LGR5^high^ and SOX9^low^ active ISC maintain homeostasis by giving rise to LGR5^low^ and SOX9^high^ reserve ISC that can differentiate into Paneth cells, supporting *Lgr5* cell stemness [24]. In the colon, SOX9 expression reveals a gradient from the crypt base to the surface epithelium: SOX9^high^ slow-dividing cells, located primarily at the crypt base, exhibit stem cell-like potential, whereas SOX9^low^ rapid-dividing cells, mainly in the TA zone, act as progenitor cells and may respond more actively to stress [23]. Furthermore, SOX9^low^ cells modulate stem and daughter cell capabilities in a Wnt-dependent manner but can differentiate into enteroendocrine cells independent of Wnt signaling [33]. Although we did not observe a dose-dependent response in gene expression of Wnt signaling pathways, the Wnt-related gene *Wnt6* correlated with *Sox9*. Gene expression of the Wnt pathway inhibitor *Bmp2* was also correlated with *Sox9*. These findings suggest that the LGR5-SOX9 stemness and differentiation systems, modulated by Wnt and BMP signaling, contribute to mucosal healing in our chronic colitis model.

The primary goal of this study was to elucidate the contributions of stem cell systems and MALT to mucosal healing. Pathological observations revealed a dose-dependent increase in MALT levels in the regenerative colon. Furthermore, we identified three distinct types of MALT—LIF, CP, and tLT—based on their morphological characteristics [25,26]. We also noted differences in the populations of each MALT type between the control and treatment groups. ILF serves as a multifunctional lymphoid nodule, playing a key role in immune development and regeneration, as well as in maintaining mucosal homeostasis in the colon [26]. The CP, which is analogous to Peyer’s patches in the small intestine, is essential for coordinating mucosal immune responses [11]. Conversely, tLTs represent newly induced lymphoid structures that emerge in response to chronic inflammation [25], and share structural similarities with mature ILF [11]. As the healthy adult colon contains pre-existing lymphoid tissues, such as the ILF and CP, distinguishing newly formed tLT in inflamed colonic tissues remains challenging [25]. In the colon tissues from patients with UC, the precise mechanisms by which lymphoid aggregates transition into ILF or inflammation-induced tLT remain unclear [26]. The formation of lymph nodes and Peyer’s plates depended on lymphoid tissue induction (LTi) and associated cytokines, such as *Cxcl13* and *Ccl20* [27]; however, the gene expression of LTi-related cytokines did not show a clear dose-dependent manner.

In IBD, disruption of the mucosal barrier affects the intestinal microbiota, leading to abnormal mucosal immune activation and exacerbation of inflammation [1,5]. CD is primarily driven by excessive Th1 and Th17 responses, whereas UC is characterized by a Th2 immune profile, with increased IL-13 levels in the colonic mucosa compared with those in patients with CD and healthy controls [34]. Although Th1 cell dysfunction is unlikely to be the initial cause of IBD, a dysregulated Th2 cell response may perpetuate chronic inflammation. In this context, CD4+CD25+ Tregs play a central role in suppressing both Th1 and Th2 immune responses [35], and are critical regulators of intestinal inflammation in the lamina propria [36]. FOXP3+ Tregs, which are abundant in the inflamed gut of patients with IBD, exhibit immunosuppressive activity in in vitro studies [36]. In the present study, we did not observe significant differences in the number of FOXP3+ Tregs in MALT across groups. However, dose–response changes and their correlation with mucosal regeneration suggested that Treg-mediated immunosuppression might contribute to mucosal healing. This hypothesis is supported by gene expression analysis, which demonstrated a significant increase in *Tgfb1* but not in *Il10* or *Il35* (*Il12a* and *Ebi3*) in the high-dose group. Certain antigen-specific TGFβ-secreting T cells (Th3 cells) may differentiate into TGFβ-inducible FOXP3+ Tregs [37,38]. TGFβ production from colonic epithelial cells, often induced by *Clostridium* spp., promotes the population of Tregs and subsequent IL-10 production [39]. TGFβ-mediated Tregs exhibit colitis-suppressing properties and therapeutic effects in chronic enteritis [40], likely facilitating mucosal regeneration in our model.

To explore the role of Tregs in colitis healing, we examined the correlation between Treg-associated gene expression and that of other essential genes. A particularly strong correlation was noted between the *Tgfβ1*, an inhibitory cytokine secreted by Tregs, and *Cxcl13*, a chemokine involved in CP and tLT formation (Figure 6G) [41]. This indicates a close interaction between Treg-mediated immunosuppression and the development of MALT in chronic colitis. Further analysis demonstrated a robust correlation between the gene expression levels of *Lgr5* and *Sox9*, and between LGR5-positive cells and lymph follicle score. This finding aligns with previous studies that have highlighted the functional interplay between these two stem cell markers [24,32]. Furthermore, the strong correlation between *Lgr5*, *Sox9*, and *Ccl20*, a chemokine involved in tLT formation, suggests an interaction between mucosal regeneration and MALT development. Collectively, these findings indicated that mucosal regeneration, immunosuppression, and MALT formation are interdependent processes that contribute to the resolution of chronic colitis.

The significant correlation between mucosal regeneration and lymph follicle scores further supported this conclusion. However, detailed pathological analysis of MALT revealed additional insights. As shown in, we identified two distinct crypt responses to tLT: crypts that extended to avoid tLT and those that infiltrated tLT. A key observation was that the CBC on the side not bordering the tLT was positive for stem cell and cell proliferation markers, whereas those on the side bordering the tLT were negative. These findings indicate three crucial points: (1) tLT may inhibit the maintenance of crypt stem cells in chronic inflammation, likely because of the localized immune environment; (2) crypt stem cells not in direct contact with tLT may compensate for this inhibition, promoting crypt stemness for mucosal regeneration; and (3) tLTs may facilitate the differentiation of CBC into mature intestinal epithelial cells during the healing process following chronic inflammation. Interestingly, the regions within the tLT where crypt stem cell activity may be altered corresponded to T-cell-dense areas enriched for FOXP3-positive Tregs. This spatial association suggests a potential interaction between Tregs and crypt stem cells within the local microenvironment, contributing to crypt homeostasis and mucosal repair. Further investigation is required to elucidate the complex interplay between tLT and regenerative crypts, with a particular focus on how TREG-derived immune signals modulate stem cell dynamics and mucosal regeneration.

This study was limited to two cycles of DSS treatment and a withdrawal protocol in mice, which may not fully replicate the chronic disease progression observed in human UC. Despite this limitation, our model successfully revealed adaptive responses of crypt stem cells and lymph follicle phenotypes, including their association with Tregs during mucosal healing. In the future, Treg activation and inhibition may be required to elucidate the role of Treg cells in mucosal regeneration in the colitis model mice. Immunohistochemical analysis of stem cell markers was confined to LGR5 and SOX9. Further studies incorporating additional colon stem cell markers are required to gain a more comprehensive understanding of stem cell systems in colitis [32,42]. Furthermore, gene expression analysis should be improved, as inconsistencies such as insufficient dose correlation and high inter-individual variability might be affected by the timing of mucosal tissue collection. Lymph follicle responses and mucosal regeneration were observed under an optical microscope; further investigations using immunological techniques are required.

In conclusion, our study successfully established a chronic colitis model with relapsing and remitting features of human UC within a relatively short experimental period. We showed that mucosal regeneration was closely correlated with the expression of crypt stem cell markers and with increased Tregs and MALT formation. These results indicate the importance of crypt stem cell activity and the immune regulatory responses mediated by Tregs and MALT as promising therapeutic targets in UC. Understanding these interactions will provide deeper insight into the relationship between acquired immunity and tissue repair mechanisms in chronic colitis.

## 4. Materials and Methods

### 4.1. Animal Study

In Experiment I, a total of 20 female 5-week-old Balb/c mice were purchased from Japan SLC Corporation (Hamamatsu, Shizuoka, Japan) and maintained under the following conditions: temperature 23 ± 3 °C, humidity 50 ± 20%, and 12 h light/12 h dark lighting cycle. We selected female BALB/c mice, as this strain is suitable for studying mucosal regeneration in colitis [8,9,30,43,44,45]. The mice were housed in clean racks with a maximum of four mice per cage using polycarbonate circular enrichment and paper-type enrichment. The animals were provided free access to rodent chow (MF; Oriental Yeast Co., Ltd., Itabashi, Tokyo, Japan) and pure water. The acclimatization period was set at 5 days for the preliminary study and 11 days for the main study. DSS (molecular weight 36,000–50,000, Cat. 160110, CAS number 9011-18-1) was purchased from MP Biomedicals (Santa Ana, CA, USA). In the preliminary study, each mouse was administered DSS at 1%, 2%, 3%, or 4% concentrations in drinking water for 5 days, followed by a 5-day withdrawal period to elucidate the dose–response to DSS. After analyzing the disease activity score (DAI) based on body weight loss, diarrhea, and fecal blood as described below, 3%, which mildly affected DAI, was selected as the high dose, and half of that amount (1.5%) was set as the low dose in the main study. In the main study, 6-week-old females were divided into three groups—control (*n* = 4; body weight, 16.8 ± 1.2 g), low-dose DSS (*n* = 6; 17.5 ± 0.9 g), and high-dose DSS (*n* = 6; 17.0 ± 0.8 g)—at the start of the study. To reduce the total number of mice, the control group comprised 4 mice, while the two treatment groups each comprised 6 mice. These mice were randomly assigned to each group based on body weight. Two cycles of DSS treatment were given with one cycle of administration of 1.5% (low-dose) and 3% (high-dose) DSS in water for 5 days, followed by a 5-day withdrawal (Supplemental Figure S17A) [6,7]. In the study, food and water intakes were measured every three days (Supplemental Figure S17B,C). Twenty days after the DSS treatments, all animals were sacrificed under isoflurane anesthesia by blood release from the aorta and vena cava, and the digestive tract and spleen were collected at autopsy. The large intestine was sampled by excising the ileocecum from the digestive tract. The distance from the colon to the anus was also measured. After measuring length, weight was determined [30,43]. For the histopathological examination and immunohistochemical staining, the colon and spleen were fixed in 4% paraformaldehyde phosphate buffer (PFA) for histopathology and immunohistochemistry.

In Experiment II, 16 female 5-week-old Balb/c mice (Japan SLC Corporation, Hamamatsu, Shizuoka, Japan) were used following the same rearing conditions and DSS treatment as Experiment I (control, *n* = 6; low-dose group, *n* = 6; high-dose group, *n* = 6) (Supplemental Figure S17A), and the colonic mucosa was collected for gene expression analysis. After 20 days of the study, all animals were sacrificed as described in Experiment I, and the mucosa of the colon was collected by scraping the visceral surface of the colon of each animal and stored at −70 °C.

The animal experimental protocols for both studies were reviewed and approved by the TUAT Experimental Animal Committee (approval numbers: R05-191 for Experiment I, approved on 10 November 2023; and R06-68 for Experiment II, approved on 25 March 2024). This study was conducted in compliance with the ARRIVE (Animal Research: Reporting of In Vivo Experiments) guidelines.

### 4.2. DAI

The DAI based on body weight loss, diarrhea, and fecal blood was analyzed using previously reported criteria (Supplemental Table S11) [44,45]. Body weights were measured at Day 1 and Day 3 of DSS administration and on the first and second days of withdrawal in each cycle. For diarrhea and fecal blood, feces were collected from three mice in each group every cycle (every 5 days). We determined the fecal score and blood score macroscopically, and the blood score was also measured using Urostic (Uropaper^®^ III Eiken, Eiken Chemical Co., Ltd., Chiyoda, Tokyo, Japan).

### 4.3. Histopathology

The PFA-fixed colon and spleen were embedded in paraffin, sectioned to a thickness of approximately 3 μm and stained with hematoxylin and eosin (HE) solution. Both tissues were examined under a microscope using a vertical slide system (Olympus, Tokyo, Japan). The lesions in the colon were scored according to previously established criteria [30,45]. for mucosal loss, inflammation, edema, and regenerative crypts (Supplemental Table S12). The total score was then calculated. In each animal, the lymphoid follicles on two sections of the colon (approximately 4 cm from the anal side) were counted and evaluated based on the lymphoid follicle scoring system (Supplemental Table S13 and Figure S6A–H).

### 4.4. Immunohistochemistry

Immunohistochemical analysis was conducted using antibodies to a cell proliferation marker Ki-67, stem cell markers SOX9 and LGR5, cell adhesion molecules E-cadherin and β-catenin, B-lymphocyte markers CD20 and PAX-5, a T-lymphocyte marker CD3, and a Treg marker Foxp3. The antibodies, antigen retrieval methods, and antibody dilution conditions are listed in Supplemental Table S14. Immunohistochemistry was conducted following the protocol of VECTASTAIN^®^Elite ABC Kit (Vector Laboratories Inc., Burlingame, CA, USA), and immunoreaction was detected using 3,3′-diaminobenzidine/H_2_O_2_, followed by counterstaining with hematoxylin. For Ki-67, SOX9, and LGR5, we counted the total and positive cells in 50 crypts following the previously reported method [30]. For Foxp3, the number of Foxp3-positive cells per 173,250 μm^2^ (165 μm × 210 μm × 5 fields) of lymph follicles found in each mouse was measured.

### 4.5. Real-Time PCR

Gene expression analysis was performed using the primer sets listed in Supplemental Table S15. Total RNA was extracted from colonic mucosa samples of six animals in the control, low-dose, and high-dose groups using the RNeasy Mini Kit (50) (QIAGEN, Hilden, Germany). Single-stranded cDNA was synthesized from 2 μg of RNA using dithiothreitol deoxynucleoside triphosphate random primers RNaseOUT and SuperScriptTM III Reverse Transcriptase (Life Technologies, Carsbad, CA, USA). Semi-quantitative RT-PCR was performed with the Power SYBR Green PCR Master Mix (Life Technologies) on an Applied Biosystems StepOnePlus Real-Time PCR System. Primers for each gene were designed using the Primer Express software (version 3.0; Life Technologies). The mRNA expression levels of each gene were corrected for the threshold cycle (CT) value of the mouse β-actin gene as an endogenous control and calculated by the 2^−ΔΔCT^ method [46].

### 4.6. Statistical Analysis

Data were presented as means and standard deviations, or as box plots. Statistical analyses were performed on body and organ weights to determine significant differences by a multiple comparison test using the Tukey–Kramer test. Significant differences were also determined using the Mann–Whitney U test for colorectal histopathology scores (mucosal loss, edema, inflammation, and mucosal regeneration), scores and numbers of lymph follicles, and FOXP3-positive cell counts. For the statistical analysis of FOX3 expression and lymph follicle scores, samples without lymph follicles in the colon (one control and two low-dose mice) were treated as zero. Significant differences were determined using Dunnett’s test for the positive cell rates of Ki-67, LGR5, and SOX9. Bonferroni correction was employed to determine significant differences using the Mann–Whitney U test. Spearman’s rank correlation coefficients (*r*_s_) were calculated to evaluate the relationships between the positive cell rates of Ki-67, LGR5, and SOX9 and the numbers of FOXP3-positive cells, histopathology scores, and the lymph follicle score. The results of each PCR were determined using the correlation coefficient. Significant differences were defined at a level of 5% or less and 1% or less. All data from each mouse were included in the analyses above.

## Data Availability

The original contributions presented in this study are included in the article/Supplementary Materials. Further inquiries can be directed to the corresponding author.

## Supplementary Materials

The following supporting information can be downloaded at: https://www.mdpi.com/article/10.3390/ijms27020779/s1.
