# Supplementary material for: Wnt and Treg-Associated Signaling Coordinate Mucosal Regeneration and MALT Formation in a Mouse Model of Chronic Colitis
Source: Int J Mol Sci. 2026 Jan 13;27(2):779. doi: 10.3390/ijms27020779 (PMC12841416; doi:10.3390/ijms27020779)
Supplement: Supplementary file 1 [file ijms-27-00779-s001.zip › ijms-3997131-supplementary.pdf]

Wnt and Treg-Associated Signaling Coordinate Mucosal Regeneration and MALT Formation in a Mouse Model of Chronic Colitis

Nanami Watanabe[1], Mio Kobayashi[2], Tatsu Kuriki[1], Yuri Ebizuka[1], Mai Hirata[1], Rintaro Mizuguchi[3], Mio Takimoto[1], Bai Yidan[1,4], Mengyuan Luo[1,5], Mai Todoroki[1,5], Ma Suzanneth G. Lola[6], Xinyu Zou[7], Sha Jiang[7], Tetsuhito Kigata[8], Makoto Shibutani[1], Toshinori Yoshida[1*], Tsutomu Omatsu[3]

[1] Laboratory of Veterinary Pathology, Cooperative Department of Veterinary Medicine, Tokyo University of Agriculture and Technology, 3-5-8 Saiwai-cho, Fuchu-shi 183-8509, Tokyo, Japan
[2] Department of Basic Pathology, Fukushima Medical University, School of Medicine, 1 Hikarigaoka, Fukushima-shi 960-1295, Japan
[3] Center for Infectious Disease Epidemiology and Prevention Research, Faculty of Agriculture, Tokyo University of Agriculture and Technology, 3-5-8 Saiwai-cho, Fuchu-shi 183-8509, Tokyo, Japan; tomatsu@cc.tuat.ac.jp (T.O.)
[4] International Innovative Agricultural Science Course (IIAS), Tokyo University of Agriculture and Technology, 3-5-8 Saiwai-cho, Fuchu-shi 183-8509, Tokyo, Japan
[5] Cooperative Division of Veterinary Sciences, Tokyo University of Agriculture and Technology, 3-5-8 Saiwai-cho, Fuchu-shi 183-8509, Tokyo, Japan
[6] Department of Veterinary Paraclinical Sciences, College of Veterinary Medicine, University of the Philippines, Los Banos, Laguna 4031, Philippines
[7] Joint International Research Laboratory of Animal Health and Animal Food Safety, College of Veterinary Medicine, Southwest University, 2nd Tiansheng Road, Beibei District, Chongqing 400715, China
[8] Laboratory of Veterinary Anatomy, Cooperative Department of Veterinary Medicine, Tokyo University of Agriculture and Technology, 3-5-8 Saiwai-cho, Fuchu-shi 183-8509, Tokyo, Japan; kigatat@go.tuat.ac.jp
* Correspondence: yoshida7@cc.tuat.ac.jp; Tel.: +81-42-367-5874

Supplemental Table S1. Individual score in body weight loss for Experiment I

Parameter		Score	Control	DSS Low	DSS High
Body weight loss	Day 3	0	3	3	3
		1	0	0	0
		2	0	0	0
		3	0	0	0
		4	0	0	0
	Day 5	0	3	3	3
		1	0	0	0
		2	0	0	0
		3	0	0	0
		4	0	0	0
	Day 8	0	3	2	3
		1	0	1	0
		2	0	0	0
		3	0	0	0
		4	0	0	0
	Day 10	0	3	3	2
		1	0	0	1
		2	0	0	0
		3	0	0	0
		4	0	0	0
	Day 13	0	3	3	3
		1	0	0	0
		2	0	0	0
		3	0	0	0
		4	0	0	0
	Day 15	0	0	1	0
		1	3	2	2
		2	0	0	1
		3	0	0	0
		4	0	0	0
	Day 18	0	3	3	3
		1	0	0	0
		2	0	0	0

	3	0	0	0
	4	0	0	0
Day 20	0	3	1	1
	1	0	2	2
	2	0	0	0
	3	0	0	0
	4	0	0	0

The mice were treated as described in Figure S17A.

Group: Control, control group (*n*=4); DSS low, the low (1.5%)-dose DSS-treated group (*n*=6); and DSS high, the high (3.0%)-dose DSS-treated group (*n*=6).

Three randomly selected mice from each group are examined and shown for each score.

Supplemental Table S2. Individual score in diarrhea for Experiment I

Parameter		Score	Control	DSS Low	DSS High
Diarrhea	Day 5	0	3	2	3
		1	0	1	0
		2	0	0	0
		3	0	0	0
		4	0	0	0
	Day 10	0	0	3	2
		1	3	0	1
		2	0	0	0
		3	0	0	0
		4	0	0	0
	Day 13	0	3	3	3
		1	0	0	0
		2	0	0	0
		3	0	0	0
		4	0	0	0
	Day 15	0	3	3	1
		1	0	0	2
		2	0	0	0
		3	0	0	0
		4	0	0	0
	Day 18	0	3	3	3
		1	0	0	0
		2	0	0	0
		3	0	0	0
		4	0	0	0

The mice were treated as described in Figure S17A.

Group: Control, control group (*n*=4); DSS low, the low (1.5%)-dose DSS-treated group (*n*=6); and DSS high, the high (3.0%)-dose DSS-treated group (*n*=6). Three randomly selected mice from each group are examined and shown for each score.

.

Supplemental Table S3. Individual score in fecal blood for Experiment I

		Score	Control	DSS Low	DSS High
Fecal blood	Day 5	0	0	0	0
		1	2	1	0
		2	1	1	1
		3	0	1	1
		4	0	0	1
	Day 10	0	0	0	0
		1	0	0	0
		2	3	2	3
		3	0	1	0
		4	0	0	0
	Day 15	0	0	0	0
		1	1	1	0
		2	2	2	1
		3	0	0	2
		4	0	0	0
	Day 18	0	3	0	0
		1	0	0	0
		2	0	0	0
		3	0	0	0
		4	0	1	3

The mice were treated as described in Figure S17A.

Group: Control, control group (*n*=4); DSS low, the low (1.5%)-dose DSS-treated group (*n*=6); and DSS high, the high (3.0%)-dose DSS-treated group (*n*=6). Three randomly selected mice from each group are examined and shown for each score.

Supplemental Table S4. Individual score in DAI for Experiment I

Parameter		Score	Control	DSS Low	DSS High
DAI	Day 5	0	0	0	0
		1	2	1	0
		2	0	1	1
		3	1	0	1
		4	0	1	1
		5	0	0	0
	Day 10	0	0	0	0
		1	0	0	0
		2	3	2	1
		3	0	1	2
		4	0	0	0
		5	0	0	0
	Day 15	0	0	0	0
		1	0	0	0
		2	1	1	0
		3	2	2	0
		4	0	0	2
		5	0	0	1
	Day 18	0	3	0	0
		1	0	0	0
		2	0	0	0
		3	0	0	0
		4	0	1	3
		5	0	0	0

The mice were treated as described in Figure S17A.

Group: Control, control group (*n*=4); DSS low, the low (1.5%)-dose DSS-treated group (*n*=6); and DSS high, the high (3.0%)-dose DSS-treated group (*n*=6). Three randomly selected mice from each group are examined and shown for each score.

DAI, Disease activity index (see Supplemental Table S11).

Supplemental Table S5. Final body weight and organ weights at autopsy for Experiment I

Item	Control	DSS Low	DSS High	
Body weight (g)	18.96±1.43	19.31±0.81	18.61±1.19	n.s.
Colon length (mm)	88.50±2.64	83.67±7.61	82.83±7.39	n.s.
Colon weight (g)	0.53±0.11	0.53±0.08	0.56±0.07	n.s.
Spleen weight (g)	0.10±0.01	0.11±0.01	0.11±0.02	n.s.
Colon weight/Body weight	0.03±0.00	0.03±0.00	0.03±0.00	n.s.
Colon length/Body weight (mm/g)	4.68±0.26	4.33±0.28	4.47±0.43	n.s.
Spleen weight/Body weight	0.01±0.00	0.01±0.00	0.01±0.00	n.s.

The mice were treated as described in Figure S17A.

Group: Control, control group (*n*=4); DSS low, the low (1.5%)-dose DSS-treated group (*n*=6); and DSS high, the high (3.0%)-dose DSS-treated group (*n*=6).

Colon length was measured between the ileocecal area and the anus.

Abbreviation: n.s, no significant difference.

Supplemental Table S6. Final body weight and organ weights at autopsy for Experiment II

Parameter	Control	DSS Low	DSS High	
Body weight (g)	19.47±0.67	20.25±0.91	19.73±0.49	n.s.
Colon length (mm)	91.33±9.71	81.33±14.99	90.00±13.80	n.s.
Colon weight/Body weight	4.69±0.39	4.03±0.72	4.55±0.57	n.s.

The mice were treated as described in Figure S17A.

Group: Control, control group (*n*=6); DSS low, the low (1.5%)-dose DSS-treated group (*n*=6); and DSS high, the high (3.0%)-dose DSS-treated group (*n*=6).

Colon length was measured between the ileocecal area and the anus.

Abbreviation: n.s, no significant difference.

Supplemental Table S7. Experiment I. Spearman's rank correlation analysis in cell proliferation markers and histopathological scores.

Spearman's rank correlation coefficient

	Ki-67	LGR5	SOX9	FOXP3	Ml	In	Ed	Mr	Ly
Ki-67	1.00	0.84	0.11	0.04	-0.05	0.08	0.41	0.48	0.55
LGR5	0.84	1.00	0.30	0.14	-0.16	0.28	0.22	0.48	0.57
SOX9	0.11	0.30	1.00	-0.15	-0.46	-0.21	-0.11	-0.06	-0.10
FOXP3	0.04	0.14	-0.15	1.00	0.31	0.77	0.18	0.74	0.10
Ml	-0.05	-0.16	-0.46	0.31	1.00	0.30	0.20	0.18	-0.38
In	0.08	0.28	-0.21	0.77	0.30	1.00	0.24	0.56	0.22
Ed	0.41	0.22	-0.11	0.18	0.20	0.24	1.00	0.43	0.45
Mr	0.48	0.48	-0.06	0.74	0.18	0.56	0.43	1.00	0.36
Ly	0.55	0.57	-0.10	0.10	-0.38	0.22	0.45	0.36	1.00

p value

	Ki-67	LGR5	SOX9	FOXP3	Ml	In	Ed	Mr	Ly
Ki-67	-	0.00	0.69	0.88	0.84	0.77	0.11	0.06	0.03
LGR5	0.00	-	0.25	0.61	0.55	0.30	0.42	0.06	0.02
SOX9	0.69	0.25	-	0.59	0.07	0.44	0.68	0.81	0.70
FOXP3	0.88	0.61	0.59	-	0.24	0.00	0.50	0.00	0.70
Ml	0.84	0.55	0.07	0.24	-	0.26	0.46	0.50	0.15
In	0.77	0.30	0.44	0.00	0.26	-	0.38	0.02	0.42
Ed	0.11	0.42	0.68	0.50	0.46	0.38	-	0.10	0.08
Mr	0.06	0.06	0.81	0.00	0.50	0.02	0.10	-	0.16
Ly	0.03	0.02	0.70	0.70	0.15	0.42	0.08	0.16	-

Statistical difference

	Ki-67	LGR5	SOX9	FOXP3	Ml	In	Ed	Mr	Ly
Ki-67	-	[**]	[ ]	[ ]	[ ]	[ ]	[ ]	[ ]	[*]
LGR5	[**]	-	[ ]	[ ]	[ ]	[ ]	[ ]	[ ]	[*]
SOX9	[ ]	[ ]	-	[ ]	[ ]	[ ]	[ ]	[ ]	[ ]
FOXP3	[ ]	[ ]	[ ]	-	[ ]	[**]	[ ]	[**]	[ ]
Ml	[ ]	[ ]	[ ]	[ ]	-	[ ]	[ ]	[ ]	[ ]
In	[ ]	[ ]	[ ]	[**]	[ ]	-	[ ]	[*]	[ ]
Ed	[ ]	[ ]	[ ]	[ ]	[ ]	[ ]	-	[ ]	[ ]
Mr	[ ]	[ ]	[ ]	[**]	[ ]	[*]	[ ]	-	[ ]

Ly	[*]	[*]	[ ]	[ ]	[ ]	[ ]	[ ]	[ ]	-

n=16.

Ml, Mucosal loss score; In, Inflammation score; Ed, Edema score; Mr, Mucosal regeneration score; Ly, Lymph follicle score.

* $p<0.05$, ** $p<0.01$.

Supplemental Table S8. Experiment II. Relative gene expression by RT-PCR

Genes	Control	DSS Low		DSS High	
	n=6	*n*=6		*n*=6	
Axin2	1.278±0.43	1.33±0.62	n.s.	0.90±0.58	n.s.
Bmi1	1.37±0.88	0.99±0.20	n.s.	0.93±0.48	n.s.
Bmp2	1.25±0.55	1.28±1.02	n.s.	1.08±0.71	n.s.
Bmp5	1.98±2.77	0.96±0.81	n.s.	2.96±3.91	n.s.
Bmp7	2.55±3.79	0.88±0.82	n.s.	2.25±2.50	n.s.
cMyc	1.38±0.33	1.04±0.52	n.s.	0.88±0.45	n.s.
Cxcl13	1.52±1.22	0.75±0.45	n.s.	2.44±2.77	n.s.
Dkk3	2.36±3.44	0.84±0.72	n.s.	2.71±4.15	n.s.
Dll1	1.69±1.28	0.96±0.54	n.s.	1.15±1.09	n.s.
Dll4	1.67±1.28	1.04±0.21	n.s.	0.90±0.55	n.s.
Ebi3	2.65±1.28	0.95±0.58	n.s.	1.12±1.40	n.s.
Grem1	2.22±2.07	0.65±0.28	n.s.	1.33±1.12	n.s.
Il12a	1.55±1.93	0.87±0.44	n.s.	1.94±1.48	n.s.
Rspo1	2.86±3.03	0.85±0.49	n.s.	1.05±1.05	n.s.
Tnfα	2.34±1.49	1.23±1.16	n.s.	2.60±2.06	n.s.
Wif1	1.00±0.23	1.15±0.60	n.s.	1.10±0.49	n.s.
Wnt2b	3.14±4.89	1.06±0.97	n.s.	6.95±14.75	n.s.
Wnt4	2.26±2.75	0.89±0.63	n.s.	2.43±4.48	n.s.
Wnt6	3.08±3.49	0.85±0.45	n.s.	1.11±1.13	n.s.

The relative mRNA expression level of each gene is shown by normalizing mouse *actb* level.

The data presented means and standard deviations.

Abbreviations: n.s., no significant difference.

The data, except for the genes shown in Figure 5, are presented.

Group: Control, control group; DSS low, the low-dose DSS-treated group; and DSS high, the high-dose DSS-treated group.

Supplemental Table S9-1. Experiment II. Individual data of gene expression by RT-PCR

Group	ID	*Axin2*	*Bmi1*	*Bmp2*	*Bmp5*	*Bmp7*	*Ccl20*	*cMyc*	*Cxcl13*	*Dkk3*	*Dll1*	*Dll4*	*Ebi3*
Control	C1	1.16	0.66	0.74	7.49	7.73	0.61	1.29	1.04	9.31	2.09	1.73	1.62
	C2	1.25	1.30	2.16	1.12	0.53	1.60	1.27	2.22	1.85	1.43	1.17	1.75
	C3	1.13	1.19	0.98	0.31	0.41	0.65	1.22	0.33	0.41	1.36	1.46	1.79
	C4	1.84	0.95	0.74	0.23	2.69	0.97	1.44	1.46	0.78	0.70	0.80	0.89
	C5	1.71	3.12	1.55	1.98	0.80	1.76	2.00	3.58	1.19	4.02	4.16	9.08
	C6	0.65	0.99	1.30	0.76	0.66	0.69	1.04	0.49	0.61	0.50	0.71	0.75
DSS Low	L1	1.41	1.14	1.58	0.22	0.32	2.17	1.49	0.43	0.40	1.41	1.27	1.79
	L2	1.25	0.95	0.37	2.02	2.41	0.99	0.71	1.58	1.53	0.36	0.80	0.26
	L3	0.51	0.82	0.43	0.30	0.48	0.79	0.68	0.66	0.19	0.86	1.11	1.34
	L4	2.40	1.31	3.11	1.93	0.94	2.19	1.82	0.64	1.95	1.80	1.26	1.10
	L5	1.42	0.92	0.83	0.67	0.73	0.73	1.04	0.30	0.37	0.61	0.89	0.41
	L6	0.91	0.79	1.32	0.62	0.47	1.33	0.48	0.90	0.62	0.75	0.88	0.77
DSS High	H1	0.27	0.79	0.39	0.81	0.84	0.54	0.50	0.54	0.76	0.46	0.82	0.66
	H2	0.18	0.52	0.40	4.85	4.47	0.45	0.48	6.90	2.55	0.62	0.16	0.38
	H3	1.17	0.71	0.85	0.48	0.96	1.41	0.87	0.74	0.37	0.83	1.41	0.95
	H4	0.83	0.84	1.07	0.62	0.62	0.55	0.76	1.06	0.80	0.60	0.48	0.44
	H5	1.59	1.88	2.20	10.17	6.08	1.99	1.71	4.94	11.0	3.34	1.64	3.94
	H6	1.39	0.84	1.58	0.84	0.26	0.98	0.93	0.49	0.76	1.02	0.89	0.36

Group: Control, control group (n=6); DSS low, the low (1.5%)-dose DSS-treated group (n=6); and DSS high, the high (3.0%)-dose DSS-treated group (n=6).
ID: Animal identification number
Each mRNA level was normalized to the actb level, the endogenous control in the same sample.

Supplemental Table S9-2. Experiment II. Individual data of gene expression by RT-PCR

Group	ID	*Foxp3*	*Grem1*	*Il10*	*Il12a*	*Lgr5*	*Rspo1*	*Sox9*	*Tgfb1*	*Tnfa*	*Wif1*	*Wnt2b*	*Wnt4*
Control	C1	1.62	2.66	1.80	0.71	0.93	2.16	0.85	1.08	0.74	1.16	12.96	7.81
	C2	1.94	1.51	1.64	1.68	2.06	1.83	1.45	0.94	2.48	0.88	1.76	1.50
	C3	2.65	2.14	1.98	0.24	1.17	3.32	2.14	0.34	1.11	0.69	0.19	0.53
	C4	0.88	0.55	0.77	0.99	2.55	0.74	0.63	1.09	3.64	0.88	0.91	0.96
	C5	7.40	6.05	9.71	5.34	3.13	8.68	1.34	1.16	4.50	1.10	0.52	1.52
	C6	0.55	0.41	0.63	0.35	1.61	0.45	1.24	0.72	1.56	1.31	2.48	1.22
DSS Low	L1	2.20	0.74	1.33	0.29	1.64	1.53	1.01	0.69	0.92	0.48	0.18	0.30
	L2	0.33	0.44	0.22	1.28	0.73	0.25	0.83	1.34	0.08	2.24	1.69	1.08
	L3	1.15	0.66	1.10	1.43	0.49	1.31	0.58	0.58	2.74	1.31	0.38	0.23
	L4	0.90	1.15	1.38	0.55	3.31	0.83	1.77	0.85	0.04	1.07	2.70	1.95
	L5	0.64	0.43	0.52	0.98	1.09	0.54	1.13	0.83	2.50	0.87	0.90	1.04
	L6	0.44	0.46	0.83	0.67	0.92	0.57	1.07	0.58	1.11	0.91	0.49	0.73
DSS High	H1	0.62	0.77	0.62	1.38	0.16	0.59	0.40	0.57	3.92	1.29	0.10	0.80
	H2	0.56	1.13	0.39	3.22	0.73	0.56	0.61	3.85	4.89	0.38	2.94	0.59
	H3	1.45	0.90	0.97	0.98	0.70	1.27	0.81	1.16	0.06	1.70	0.33	0.40
	H4	0.30	0.77	0.78	1.44	0.76	0.32	0.72	1.02	3.72	0.82	0.88	0.73
	H5	2.28	3.61	2.55	4.25	1.22	3.08	2.06	3.23	0.06	1.53	37.0	11.6
	H6	0.45	0.79	0.41	0.36	0.23	0.50	1.16	1.71	2.94	0.89	0.47	0.50

Group: Control, control group (*n*=6); DSS low, the low (1.5%)-dose DSS-treated group (*n*=6); and DSS high, the high (3.0%)-dose DSS-treated group (*n*=6).
ID: Animal identification number
Each mRNA level was normalized to the actb level, the endogenous control in the same sample.

Supplemental Table S10-1. Experiment II. Spearman's rank correlation analysis in gene expression.

Spearman's rank correlation coefficient

n=18

	Axin2	*Bmi1*	*Bmp2*	*Bmp5*	*Bmp7*	*Ccl20*	*cMyc*	*Cxcl13*	*Dkk3*	*Dll1*	*Dll4*	*Ebi3*	*Foxp3*
Axin2	1.00	0.66	0.53	0.08	0.14	0.74	0.80	0.05	0.22	0.50	0.46	0.33	0.37
Bmi1	0.66	1.00	0.66	0.09	-0.17	0.66	0.75	0.02	0.18	0.47	0.40	0.54	0.48
Bmp2	0.53	0.66	1.00	0.07	-0.34	0.70	0.59	-0.04	0.19	0.64	0.42	0.47	0.33
Bmp5	0.08	0.09	0.07	1.00	0.59	0.00	0.14	0.56	0.83	0.23	0.12	-0.07	0.01
Bmp7	0.14	-0.17	-0.34	0.59	1.00	-0.11	0.15	0.58	0.60	0.01	0.01	-0.03	0.06
Ccl20	0.74	0.66	0.70	0.00	-0.11	1.00	0.59	0.10	0.07	0.58	0.52	0.50	0.42
cMyc	0.80	0.75	0.59	0.14	0.15	0.59	1.00	0.03	0.30	0.72	0.66	0.68	0.70
Cxcl13	0.05	0.02	-0.04	0.56	0.58	0.10	0.03	1.00	0.71	0.19	-0.04	0.10	0.04
Dkk3	0.22	0.18	0.19	0.83	0.60	0.07	0.30	0.71	1.00	0.33	0.06	0.07	0.03
Dll1	0.50	0.47	0.64	0.23	0.01	0.58	0.72	0.19	0.33	1.00	0.87	0.82	0.79
Dll4	0.46	0.40	0.42	0.12	0.01	0.52	0.66	-0.04	0.06	0.87	1.00	0.82	0.86
Ebi3	0.33	0.54	0.47	-0.07	-0.03	0.50	0.68	0.10	0.07	0.82	0.82	1.00	0.91
Foxp3	0.37	0.48	0.33	0.01	0.06	0.42	0.70	0.04	0.03	0.79	0.86	0.91	1.00
Grem1	0.21	0.27	0.37	0.48	0.24	0.20	0.49	0.42	0.55	0.79	0.69	0.62	0.67
Il10	0.32	0.51	0.52	0.05	-0.01	0.46	0.67	0.13	0.17	0.86	0.85	0.95	0.84
Il12a	-0.05	0.01	-0.15	0.45	0.46	-0.03	-0.05	0.81	0.44	0.07	-0.05	0.08	0.08
Lgr5	0.64	0.74	0.57	0.03	0.08	0.52	0.81	0.11	0.29	0.51	0.33	0.58	0.49
Rspo1	0.28	0.38	0.31	0.03	0.06	0.37	0.60	0.14	0.11	0.84	0.87	0.93	0.96
Sox9	0.50	0.77	0.76	0.25	-0.21	0.52	0.62	-0.12	0.22	0.58	0.53	0.47	0.45
Tgfb1	0.30	-0.03	0.06	0.59	0.55	0.15	0.14	0.67	0.59	0.14	-0.03	-0.21	-0.09
Tnfa	-0.36	-0.25	-0.33	-0.10	-0.16	-0.56	-0.30	0.15	-0.08	-0.30	-0.43	-0.28	-0.21
Wif1	0.01	0.02	-0.12	0.31	0.37	0.22	-0.05	0.16	0.03	-0.04	0.18	0.02	-0.03
Wnt2b	0.21	0.15	0.15	0.71	0.65	0.04	0.28	0.54	0.77	0.18	-0.07	-0.03	-0.05
Wnt4	0.42	0.44	0.26	0.72	0.58	0.20	0.53	0.39	0.73	0.30	0.22	0.22	0.17
Wnt6	0.33	0.46	0.50	0.15	0.00	0.51	0.60	0.05	0.14	0.83	0.93	0.87	0.84

continued

	Grem1	*Il10*	*Il12a*	*Lgr5*	*Rspo1*	*Sox9*	*Tgfb1*	*Tnfa*	*Wif1*	*Wnt2b*	*Wnt4*	*Wnt6*
Axin2	0.21	0.32	-0.05	0.64	0.28	0.50	0.30	-0.36	0.01	0.21	0.42	0.33
Bmi1	0.27	0.51	0.01	0.74	0.38	0.77	-0.03	-0.25	0.02	0.15	0.44	0.46
Bmp2	0.37	0.52	-0.15	0.57	0.31	0.76	0.06	-0.33	-0.12	0.15	0.26	0.50
Bmp5	0.48	0.05	0.45	0.03	0.03	0.25	0.59	-0.10	0.31	0.71	0.72	0.15

Bmp7	0.24	-0.01	0.46	0.08	0.06	-0.21	0.55	-0.16	0.37	0.65	0.58	0.00
Ccl20	0.20	0.46	-0.03	0.52	0.37	0.52	0.15	-0.56	0.22	0.04	0.20	0.51
cMyc	0.49	0.67	-0.05	0.81	0.60	0.62	0.14	-0.30	-0.05	0.28	0.53	0.60
Cxcl13	0.42	0.13	0.81	0.11	0.14	-0.12	0.67	0.15	0.16	0.54	0.39	0.05
Dkk3	0.55	0.17	0.44	0.29	0.11	0.22	0.59	-0.08	0.03	0.77	0.73	0.14
Dll1	0.79	0.86	0.07	0.51	0.84	0.58	0.14	-0.30	-0.04	0.18	0.30	0.83
Dll4	0.69	0.85	-0.05	0.33	0.87	0.53	-0.03	-0.43	0.18	-0.07	0.22	0.93
Ebi3	0.62	0.95	0.08	0.58	0.93	0.47	-0.21	-0.28	0.02	-0.03	0.22	0.87
Foxp3	0.67	0.84	0.08	0.49	0.96	0.45	-0.09	-0.21	-0.03	-0.05	0.17	0.84
Grem1	1.00	0.72	0.33	0.25	0.74	0.41	0.31	-0.07	-0.06	0.21	0.33	0.72
Il10	0.72	1.00	0.09	0.55	0.90	0.55	-0.17	-0.32	0.05	0.06	0.32	0.90
Il12a	0.33	0.09	1.00	-0.05	0.13	-0.26	0.47	0.40	0.17	0.30	0.29	-0.01
Lgr5	0.25	0.55	-0.05	1.00	0.43	0.60	-0.03	-0.20	-0.24	0.38	0.54	0.39
Rspo1	0.74	0.90	0.13	0.43	1.00	0.40	-0.13	-0.19	-0.01	-0.05	0.18	0.89
Sox9	0.41	0.55	-0.26	0.60	0.40	1.00	-0.01	-0.44	-0.03	0.26	0.46	0.60
Tgfb1	0.31	-0.17	0.47	-0.03	-0.13	-0.01	1.00	0.00	0.15	0.54	0.27	-0.12
Tnfa	-0.07	-0.32	0.40	-0.20	-0.19	-0.44	0.00	1.00	-0.44	-0.23	-0.25	-0.40
Wif1	-0.06	0.05	0.17	-0.24	-0.01	-0.03	0.15	-0.44	1.00	0.14	0.31	0.23
Wnt2b	0.21	0.06	0.30	0.38	-0.05	0.26	0.54	-0.23	0.14	1.00	0.75	-0.05
Wnt4	0.33	0.32	0.29	0.54	0.18	0.46	0.27	-0.25	0.31	0.75	1.00	0.31
Wnt6	0.72	0.90	-0.01	0.39	0.89	0.60	-0.12	-0.40	0.23	-0.05	0.31	1.00

Supplemental Table S10-2. Experiment II. Spearman's rank correlation analysis in gene expression.

p value

n=18

	Axin2	*Bmi1*	*Bmp2*	*Bmp5*	*Bmp7*	*Ccl20*	*cMyc*	*Cxcl13*	*Dkk3*	*Dll1*	*Dll4*	*Ebi3*	*Foxp3*
Axin2	-	0.00	0.02	0.74	0.59	0.00	0.00	0.85	0.38	0.03	0.06	0.18	0.13
Bmi1	0.00	-	0.00	0.71	0.50	0.00	0.00	0.93	0.46	0.05	0.10	0.02	0.04
Bmp2	0.02	0.00	-	0.79	0.16	0.00	0.01	0.88	0.46	0.00	0.08	0.05	0.19
Bmp5	0.74	0.71	0.79	-	0.01	0.99	0.57	0.02	0.00	0.35	0.64	0.80	0.98
Bmp7	0.59	0.50	0.16	0.01	-	0.65	0.55	0.01	0.01	0.98	0.96	0.91	0.82
Ccl20	0.00	0.00	0.00	0.99	0.65	-	0.01	0.70	0.79	0.01	0.03	0.03	0.09
cMyc	0.00	0.00	0.01	0.57	0.55	0.01	-	0.91	0.22	0.00	0.00	0.00	0.00
Cxcl13	0.85	0.93	0.88	0.02	0.01	0.70	0.91	-	0.00	0.45	0.87	0.69	0.88
Dkk3	0.38	0.46	0.46	0.00	0.01	0.79	0.22	0.00	-	0.19	0.82	0.79	0.89
Dll1	0.03	0.05	0.00	0.35	0.98	0.01	0.00	0.45	0.19	-	0.00	0.00	0.00
Dll4	0.06	0.10	0.08	0.64	0.96	0.03	0.00	0.87	0.82	0.00	-	0.00	0.00
Ebi3	0.18	0.02	0.05	0.80	0.91	0.03	0.00	0.69	0.79	0.00	0.00	-	0.00
Foxp3	0.13	0.04	0.19	0.98	0.82	0.09	0.00	0.88	0.89	0.00	0.00	0.00	-
Grem1	0.41	0.29	0.13	0.04	0.34	0.42	0.04	0.08	0.02	0.00	0.00	0.01	0.00
Il10	0.19	0.03	0.03	0.84	0.98	0.06	0.00	0.60	0.49	0.00	0.00	0.00	0.00
Il12a	0.85	0.96	0.56	0.06	0.05	0.90	0.84	0.00	0.07	0.79	0.83	0.76	0.75
Lgr5	0.00	0.00	0.01	0.92	0.75	0.03	0.00	0.66	0.25	0.03	0.18	0.01	0.04
Rspo1	0.26	0.12	0.20	0.89	0.81	0.13	0.01	0.59	0.66	0.00	0.00	0.00	0.00
Sox9	0.03	0.00	0.00	0.32	0.40	0.03	0.01	0.62	0.38	0.01	0.02	0.05	0.06
Tgfb1	0.22	0.91	0.82	0.01	0.02	0.56	0.59	0.00	0.01	0.59	0.91	0.41	0.72
Tnfa	0.15	0.32	0.18	0.69	0.53	0.02	0.23	0.56	0.77	0.23	0.07	0.26	0.40
Wif1	0.96	0.93	0.63	0.21	0.13	0.37	0.84	0.53	0.89	0.86	0.47	0.93	0.92
Wnt2b	0.41	0.56	0.56	0.00	0.00	0.88	0.26	0.02	0.00	0.46	0.77	0.91	0.84
Wnt4	0.08	0.07	0.30	0.00	0.01	0.43	0.02	0.11	0.00	0.23	0.38	0.38	0.49
Wnt6	0.18	0.06	0.04	0.54	0.99	0.03	0.01	0.85	0.59	0.00	0.00	0.00	0.00

continued

	Grem1	*Il10*	*Il12a*	*Lgr5*	*Rspo1*	*Sox9*	*Tgfb1*	*Tnfa*	*Wif1*	*Wnt2b*	*Wnt4*	*Wnt6*
Axin2	0.41	0.19	0.85	0.00	0.26	0.03	0.22	0.15	0.96	0.41	0.08	0.18
Bmi1	0.29	0.03	0.96	0.00	0.12	0.00	0.91	0.32	0.93	0.56	0.07	0.06
Bmp2	0.13	0.03	0.56	0.01	0.20	0.00	0.82	0.18	0.63	0.56	0.30	0.04
Bmp5	0.04	0.84	0.06	0.92	0.89	0.32	0.01	0.69	0.21	0.00	0.00	0.54

Bmp7	0.34	0.98	0.05	0.75	0.81	0.40	0.02	0.53	0.13	0.00	0.01	0.99
Ccl20	0.42	0.06	0.90	0.03	0.13	0.03	0.56	0.02	0.37	0.88	0.43	0.03
cMyc	0.04	0.00	0.84	0.00	0.01	0.01	0.59	0.23	0.84	0.26	0.02	0.01
Cxcl13	0.08	0.60	0.00	0.66	0.59	0.62	0.00	0.56	0.53	0.02	0.11	0.85
Dkk3	0.02	0.49	0.07	0.25	0.66	0.38	0.01	0.77	0.89	0.00	0.00	0.59
Dll1	0.00	0.00	0.79	0.03	0.00	0.01	0.59	0.23	0.86	0.46	0.23	0.00
Dll4	0.00	0.00	0.83	0.18	0.00	0.02	0.91	0.07	0.47	0.77	0.38	0.00
Ebi3	0.01	0.00	0.76	0.01	0.00	0.05	0.41	0.26	0.93	0.91	0.38	0.00
Foxp3	0.00	0.00	0.75	0.04	0.00	0.06	0.72	0.40	0.92	0.84	0.49	0.00
Grem1	-	0.00	0.18	0.32	0.00	0.09	0.21	0.78	0.82	0.40	0.18	0.00
Il10	0.00	-	0.73	0.02	0.00	0.02	0.49	0.19	0.85	0.82	0.19	0.00
Il12a	0.18	0.73	-	0.83	0.61	0.30	0.05	0.10	0.49	0.22	0.24	0.97
Lgr5	0.32	0.02	0.83	-	0.08	0.01	0.89	0.43	0.34	0.12	0.02	0.11
Rspo1	0.00	0.00	0.61	0.08	-	0.10	0.60	0.45	0.96	0.83	0.48	0.00
Sox9	0.09	0.02	0.30	0.01	0.10	-	0.98	0.07	0.91	0.30	0.06	0.01
Tgfb1	0.21	0.49	0.05	0.89	0.60	0.98	-	1.00	0.55	0.02	0.28	0.62
Tnfa	0.78	0.19	0.10	0.43	0.45	0.07	1.00	-	0.07	0.36	0.32	0.10
Wif1	0.82	0.85	0.49	0.34	0.96	0.91	0.55	0.07	-	0.59	0.21	0.35
Wnt2b	0.40	0.82	0.22	0.12	0.83	0.30	0.02	0.36	0.59	-	0.00	0.84
Wnt4	0.18	0.19	0.24	0.02	0.48	0.06	0.28	0.32	0.21	0.00	-	0.20
Wnt6	0.00	0.00	0.97	0.11	0.00	0.01	0.62	0.10	0.35	0.84	0.20	-

Supplemental Table S10-3. Experiment II. Spearman's rank correlation analysis in gene expression.

Statistically significant difference

n=18

	Axin2	*Bmi1*	*Bmp2*	*Bmp5*	*Bmp7*	*Ccl20*	*cMyc*	*Cxcl13*	*Dkk3*	*Dll1*	*Dll4*	*Ebi3*	*Foxp3*
Axin2	-	[**]	[*]	[ ]	[ ]	[**]	[**]	[ ]	[ ]	[*]	[ ]	[ ]	[ ]
Bmi1	[**]	-	[**]	[ ]	[ ]	[**]	[**]	[ ]	[ ]	[ ]	[ ]	[*]	[*]
Bmp2	[*]	[**]	-	[ ]	[ ]	[**]	[**]	[ ]	[ ]	[**]	[ ]	[*]	[ ]
Bmp5	[ ]	[ ]	[ ]	-	[*]	[ ]	[ ]	[*]	[**]	[ ]	[ ]	[ ]	[ ]
Bmp7	[ ]	[ ]	[ ]	[*]	-	[ ]	[ ]	[*]	[**]	[ ]	[ ]	[ ]	[ ]
Ccl20	[**]	[**]	[**]	[ ]	[ ]	-	[*]	[ ]	[ ]	[*]	[*]	[*]	[ ]
cMyc	[**]	[**]	[**]	[ ]	[ ]	[*]	-	[ ]	[ ]	[**]	[**]	[**]	[**]
Cxcl13	[ ]	[ ]	[ ]	[*]	[*]	[ ]	[ ]	-	[**]	[ ]	[ ]	[ ]	[ ]
Dkk3	[ ]	[ ]	[ ]	[**]	[**]	[ ]	[ ]	[**]	-	[ ]	[ ]	[ ]	[ ]
Dll1	[*]	[ ]	[**]	[ ]	[ ]	[*]	[**]	[ ]	[ ]	-	[**]	[**]	[**]
Dll4	[ ]	[ ]	[ ]	[ ]	[ ]	[*]	[**]	[ ]	[ ]	[**]	-	[**]	[**]
Ebi3	[ ]	[*]	[*]	[ ]	[ ]	[*]	[**]	[ ]	[ ]	[**]	[**]	-	[**]
Foxp3	[ ]	[*]	[ ]	[ ]	[ ]	[ ]	[**]	[ ]	[ ]	[**]	[**]	[**]	-
Grem1	[ ]	[ ]	[ ]	[*]	[ ]	[ ]	[*]	[ ]	[*]	[**]	[**]	[**]	[**]
Il10	[ ]	[*]	[*]	[ ]	[ ]	[ ]	[**]	[ ]	[ ]	[**]	[**]	[**]	[**]
Il12a	[ ]	[ ]	[ ]	[ ]	[ ]	[ ]	[ ]	[**]	[ ]	[ ]	[ ]	[ ]	[ ]
Lgr5	[**]	[**]	[*]	[ ]	[ ]	[*]	[**]	[ ]	[ ]	[*]	[ ]	[*]	[*]
Rspo1	[ ]	[ ]	[ ]	[ ]	[ ]	[ ]	[**]	[ ]	[ ]	[**]	[**]	[**]	[**]
Sox9	[*]	[**]	[**]	[ ]	[ ]	[*]	[**]	[ ]	[ ]	[*]	[*]	[ ]	[ ]
Tgfb1	[ ]	[ ]	[ ]	[**]	[*]	[ ]	[ ]	[**]	[*]	[ ]	[ ]	[ ]	[ ]
Tnfa	[ ]	[ ]	[ ]	[ ]	[ ]	[*]	[ ]	[ ]	[ ]	[ ]	[ ]	[ ]	[ ]
Wif1	[ ]	[ ]	[ ]	[ ]	[ ]	[ ]	[ ]	[ ]	[ ]	[ ]	[ ]	[ ]	[ ]
Wnt2b	[ ]	[ ]	[ ]	[**]	[**]	[ ]	[ ]	[*]	[**]	[ ]	[ ]	[ ]	[ ]
Wnt4	[ ]	[ ]	[ ]	[**]	[*]	[ ]	[*]	[ ]	[**]	[ ]	[ ]	[ ]	[ ]
Wnt6	[ ]	[ ]	[*]	[ ]	[ ]	[*]	[**]	[ ]	[ ]	[**]	[**]	[**]	[**]

continued

	Grem1	*Il10*	*Il12a*	*Lgr5*	*Rspo1*	*Sox9*	*Tgfb1*	*Tnfa*	*Wif1*	*Wnt2b*	*Wnt4*	*Wnt6*
Axin2	[ ]	[ ]	[ ]	[**]	[ ]	[*]	[ ]	[ ]	[ ]	[ ]	[ ]	[ ]
Bmi1	[ ]	[*]	[ ]	[**]	[ ]	[**]	[ ]	[ ]	[ ]	[ ]	[ ]	[ ]
Bmp2	[ ]	[*]	[ ]	[*]	[ ]	[**]	[ ]	[ ]	[ ]	[ ]	[ ]	[*]
Bmp5	[*]	[ ]	[ ]	[ ]	[ ]	[ ]	[**]	[ ]	[ ]	[**]	[**]	[ ]

Bmp7	[ ]	[ ]	[ ]	[ ]	[ ]	[ ]	[*]	[ ]	[ ]	[**]	[*]	[ ]
Ccl20	[ ]	[ ]	[ ]	[*]	[ ]	[*]	[ ]	[*]	[ ]	[ ]	[ ]	[*]
cMyc	[*]	[**]	[ ]	[**]	[**]	[**]	[ ]	[ ]	[ ]	[ ]	[*]	[**]
Cxcl13	[ ]	[ ]	[**]	[ ]	[ ]	[ ]	[**]	[ ]	[ ]	[*]	[ ]	[ ]
Dkk3	[*]	[ ]	[ ]	[ ]	[ ]	[ ]	[*]	[ ]	[ ]	[**]	[**]	[ ]
Dll1	[**]	[**]	[ ]	[*]	[**]	[*]	[ ]	[ ]	[ ]	[ ]	[ ]	[**]
Dll4	[**]	[**]	[ ]	[ ]	[**]	[*]	[ ]	[ ]	[ ]	[ ]	[ ]	[**]
Ebi3	[**]	[**]	[ ]	[*]	[**]	[ ]	[ ]	[ ]	[ ]	[ ]	[ ]	[**]
Foxp3	[**]	[**]	[ ]	[*]	[**]	[ ]	[ ]	[ ]	[ ]	[ ]	[ ]	[**]
Grem1	-	[**]	[ ]	[ ]	[**]	[ ]	[ ]	[ ]	[ ]	[ ]	[ ]	[**]
Il10	[**]	-	[ ]	[*]	[**]	[*]	[ ]	[ ]	[ ]	[ ]	[ ]	[**]
Il12a	[ ]	[ ]	-	[ ]	[ ]	[ ]	[ ]	[ ]	[ ]	[ ]	[ ]	[ ]
Lgr5	[ ]	[*]	[ ]	-	[ ]	[**]	[ ]	[ ]	[ ]	[ ]	[*]	[ ]
Rspo1	[**]	[**]	[ ]	[ ]	-	[ ]	[ ]	[ ]	[ ]	[ ]	[ ]	[**]
Sox9	[ ]	[*]	[ ]	[**]	[ ]	-	[ ]	[ ]	[ ]	[ ]	[ ]	[**]
Tgfb1	[ ]	[ ]	[ ]	[ ]	[ ]	[ ]	-	[ ]	[ ]	[*]	[ ]	[ ]
Tnfa	[ ]	[ ]	[ ]	[ ]	[ ]	[ ]	[ ]	-	[ ]	[ ]	[ ]	[ ]
Wif1	[ ]	[ ]	[ ]	[ ]	[ ]	[ ]	[ ]	[ ]	-	[ ]	[ ]	[ ]
Wnt2b	[ ]	[ ]	[ ]	[ ]	[ ]	[ ]	[*]	[ ]	[ ]	-	[**]	[ ]
Wnt4	[ ]	[ ]	[ ]	[*]	[ ]	[ ]	[ ]	[ ]	[ ]	[**]	-	[ ]
Wnt6	[**]	[**]	[ ]	[ ]	[**]	[**]	[ ]	[ ]	[ ]	[ ]	[ ]	-

* p<0.05, ** p<0.01.

Supplemental Table S11. Criteria of disease activity index (DAI)

Score	Body weight loss	Diarrhea	Fecal blood
0	-	normal	no blood
1	1-5%	softer	weak hemoccult positive
2	6-10%	unformed	moderate hemoccult positive
3	11-15%	diarrhea	strong hemoccult positive
4	>15%	no stool	visual blood

DAI was determined by scoring each parameter according to Kangawa et al., 2017 [43]; Sann et al., 2013 [44].

Supplemental Table S12. Criteria of histological evaluation

Score	Mucosal loss	Inflammation	Edema	Regeneration
0	none	none	none	none
1	≤25%	minimal	≥25%	≥25%
2	26-50%	mild	26-50%	26-50%
3	51-75%	moderate	51-75%	51-75%
4	≥76%	severe	≥76%	≥76%

Histopathological changes were evaluated by scoring each parameter according to Kangawa et al., 2017 [45].

Supplemental Table S13. Criteria of lymph follicle evaluation

Score	Size
0	-
1	single, small
2	single, large
3	double
4	triple

Criteria were set, based on the number and size of lymph follicles (See Figure 1 and Supplemental Figure S16)

Supplemental Table S14. Antibody used in immunohistochemistry

Antigen	Host species	Clonality	Clone	Dilution	Antigen retrieval	Manufacture
β-catenin	Rabbit	Polyclonal	n.a.	1:2000	Microwaving[a]	Abcam (Cambridge, UK)
E-cadherin	Mouse	Monoclonal	M168	1:500	Autoclaving[b]	Abcam (Cambridge, UK)
CD3	Rabbit	Polyclonal	n.a.	1:100	Microwaving[c]	Abcam (Cambridge, UK)
CD20	Rabbit	Polyclonal	n.a.	1:1000	Microwaving[a]	Invitrogen (Tokyo, Japan)
FOXP3	Rabbit	Polyclonal	EPR22102-37	1:500	Autoclaving[b]	Abcam (Cambridge, UK)
Ki-67	Rabbit	Monoclonal	MIB-1	1:500	Autoclaving[b]	Abcam (Cambridge, UK)
LGR5	Rabbit	Polyclonal	n.a.	1:150	Autoclaving[b]	Abcam (Cambridge, UK)
PAX5	Mouse	Monoclonal	24	1:100	Microwaving[c]	BD transduction Laboratories (Lexington, KY)
SOX9	Rabbit	Monoclonal	n.a.	1:2000	Microwaving[c]	Abcam (Cambridge, UK)

Abbreviations: CD3, Cluster of Differentiation 3; CD20, Cluster of Differentiation 20; FOXP3, Forkhead box P3; LGR5, leucine-rich repeat-containing G-protein-coupled-receptor; PAX5, paired box gene 5; SOX9, sex determining region Y-box transcription factor 9.

a: at 90℃ for 10 min in 10 mM citrate buffer (pH 6.0).

b: at 121℃ for 10 min in mM citrate buffer (pH 6.0)

c: at 90℃ for 10 min in Dako target retrieval solution (pH 9.0)

Supplemental Table S15. Primer sequences for real-time RT-PCR detection

Genes	Accession	Forward primers	Reverse primers
Actb	NM_007393	CGCAGCCACTGTCGAGTC	GTCATCCATGGCGAACTGGT
Axin2	NM_001417414	CAGCTGAAAACGGATTCAGG	CAGTTTCAGTTTCTCCAGCC
Bmi1	NM_001416911	ATCCCCACTTAATGTGTGTCCT	CTTGCTGGTCTCCAAGTAACG
Bmp2	NM_007553	ACCCCCAGCAAGGACGTCGT	AAGAAGCGCCGGGCCGTTTT
Bmp5	NM_007555	ATCAGGACCCCTCCAGGATGCC	TGATCCAGTCCTGCCATCCCAGATC
Bmp7	NM_007557	CAAGCAGCGCAGCCAGAATCG	CAATGATCCAGTCCTGCCAGCCAA
Ccl20	NM_016960	TTGTGGGTTTCACAAGACAGATG	TCTTCGTGTGAAAGATGATAGCATT
cMyc	NM_001177353	TTCATCTGCGATCCTGACGAC	CACTGAGGGGTCAATGCACTC
Cxcl13	XM_008542388	CATAGATVGGATTCAACTTCA	TCTTGGTCCAGACACAACTTCA
Dkk3	NM_001360260	GTACACCTGCCAGCCATG	CCTCTGGTTGTCACAGATG
Dll1	NM_007865	CCGGCTGAAGCTACAGAAAC	AGCCCCAATGATGCTAACAG
Dll4	NM_019454	CCTCTCGAACTTGGACTTGC	TGGAAATACAGATGCCCACA
Ebi3	NM_015766	CTTCTCTCTCAAGTACCGACTC	TTATGGGGTGCACTTTCTACTT
Foxp3	NM_138603	CTCATGATAGTGCCTGTGTCCTCAA	AGGGCCAGCATAGGTGCAAG
Grem1	XM_004274034	AGCCCAAGAAGTTCACCACCA	TATGCAACGGCACTGCCTCAC
Il10	NM_010548	AGGCAGCCTTGCAGAAAAGAG	AGTAAGAGCAGGCAGCATAGCA
Il12a	NM_001410419	GACCTGTTTACCACTGGAACTA	GATCTGCTGATGGTTGTGATTC
Lgr5	NM_010195	CCTACTCGAAGACTTACCCAGT	GCATTGGGGTGAATGATAGCA
Rspo1	NM_138683	AAGGAGTGGAACCTTCTGGAG	CCAATCTGCCATCCATCTGT
Sox9	AK049986	GAGCCGGATCTGAAGAGGGA	GCTTGACGTGTGGCTTGTTC
Tgfb1	NM_011577	TGATACGCCTGAGTGGCTGTCT	CACAAGAGCAGTGAGCGCTGAA
Tnfα	NM_013693	GGTGCCTATGTCTCAGCCTCTT	GCCATAGAACTGATGAGAGGGAG
Wif1	NM_011915	GATCCAACTGTCAATGTCCCTT	ACACGGGAAACCAACTTGAAC
Wnt2b	XM_062191970	GAGAAGAGGCTTAAGGATGC	ACCTGCAGCCTTGTCCAAG
Wnt4	NM_009523	ACGTGCGAGAAACTCAAAGG	GGACTGTGAGAAGGCTACGC
Wnt6	NM_009526	TGCCCGAGGCGCAAGACTG	ATTGCAAACACGAAAGCTGTCTCTC

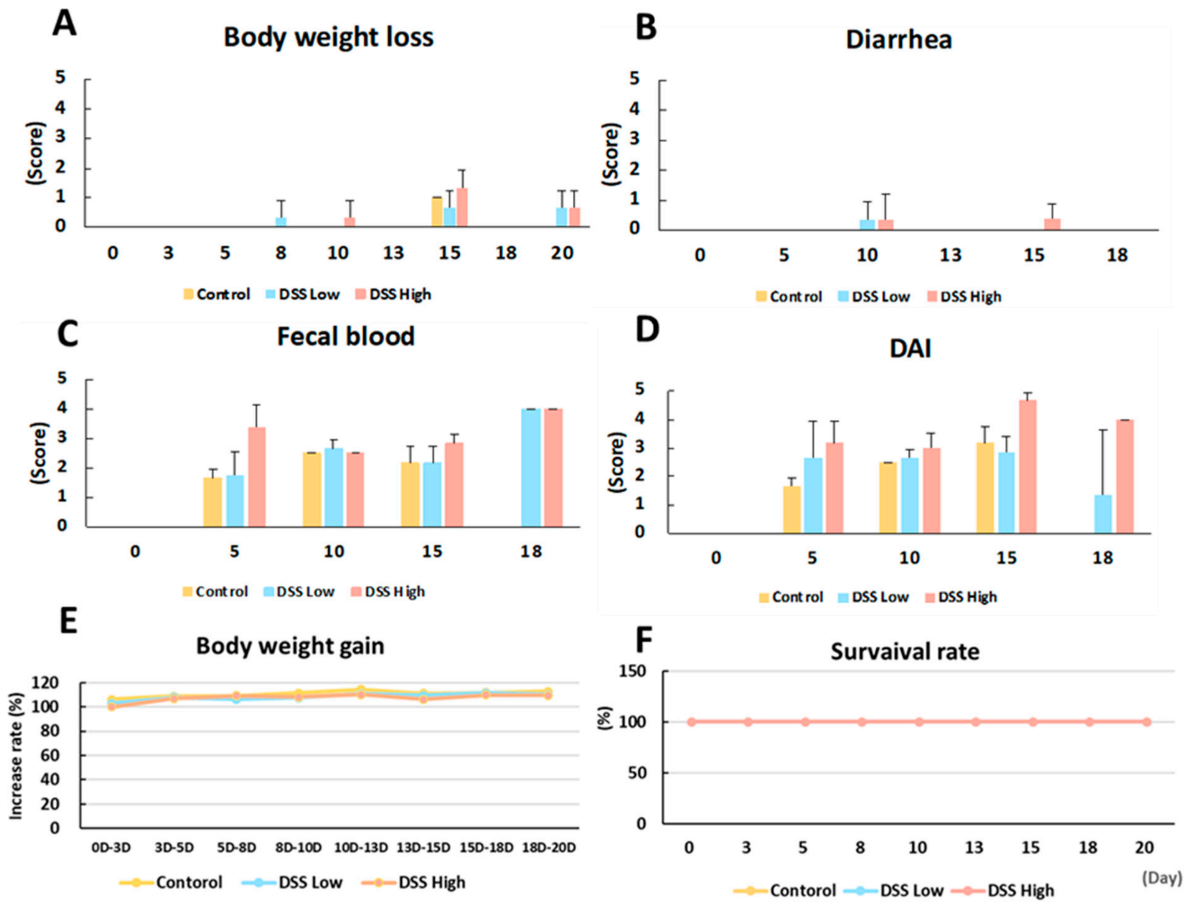

Supplemental Figure S1. Experiment I. Disease activity index (DAI) and body weight gain in control and DSS-treated mice. The control mice were exposed to pure water, and the treated mice were exposed to 1.5% and 3.0% DSS-containing water for 5 days, followed by withdrawal of DSS for 5 days (one cycle). The same treatment and withdrawal were repeated, and a total of 2 cycles were applied to the treated mice. (A) Body weight loss (score). (B) Diarrhea (score). (C) Facial blood (score). (D) DAI was calculated from scores for weight loss, diarrhea, and facial blood (total score). The data represent the mean values and standard deviations for body weight loss scores, diarrhea scores, fecal blood scores, and DAI. (E) Body weight gain (%). The data represent the mean rate of increase. (F) Survival rate (%). *n*=4, control group (Control); *n*=6, 1.5 and 3.0% DSS-treated group (DSS Low, DSS High).

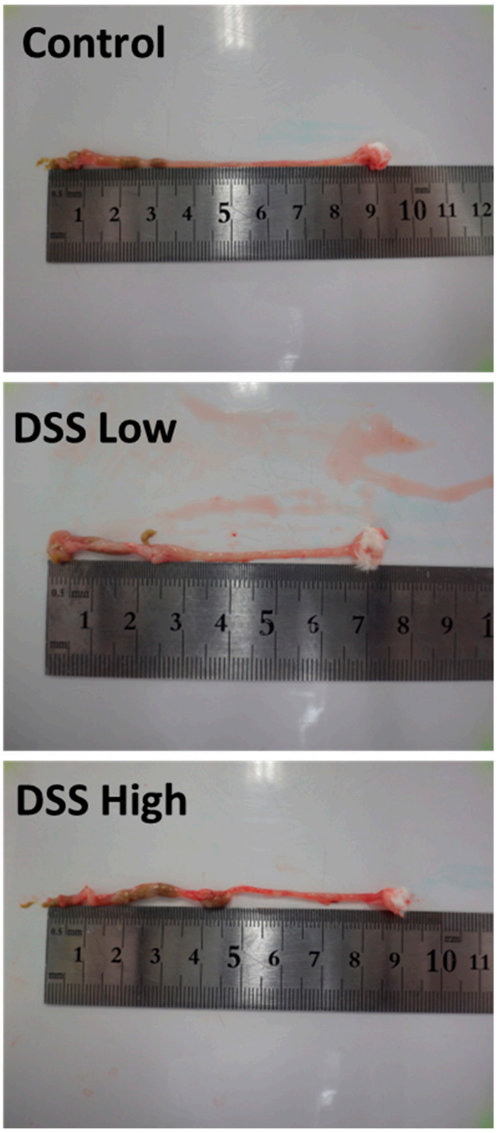

Supplemental Figure S2. Experiment I. Representative images of the colon from each group. Control, Control group; DSS Low, 1.5% DSS-treated group; DSS High, 3.0% DSS-treated group.

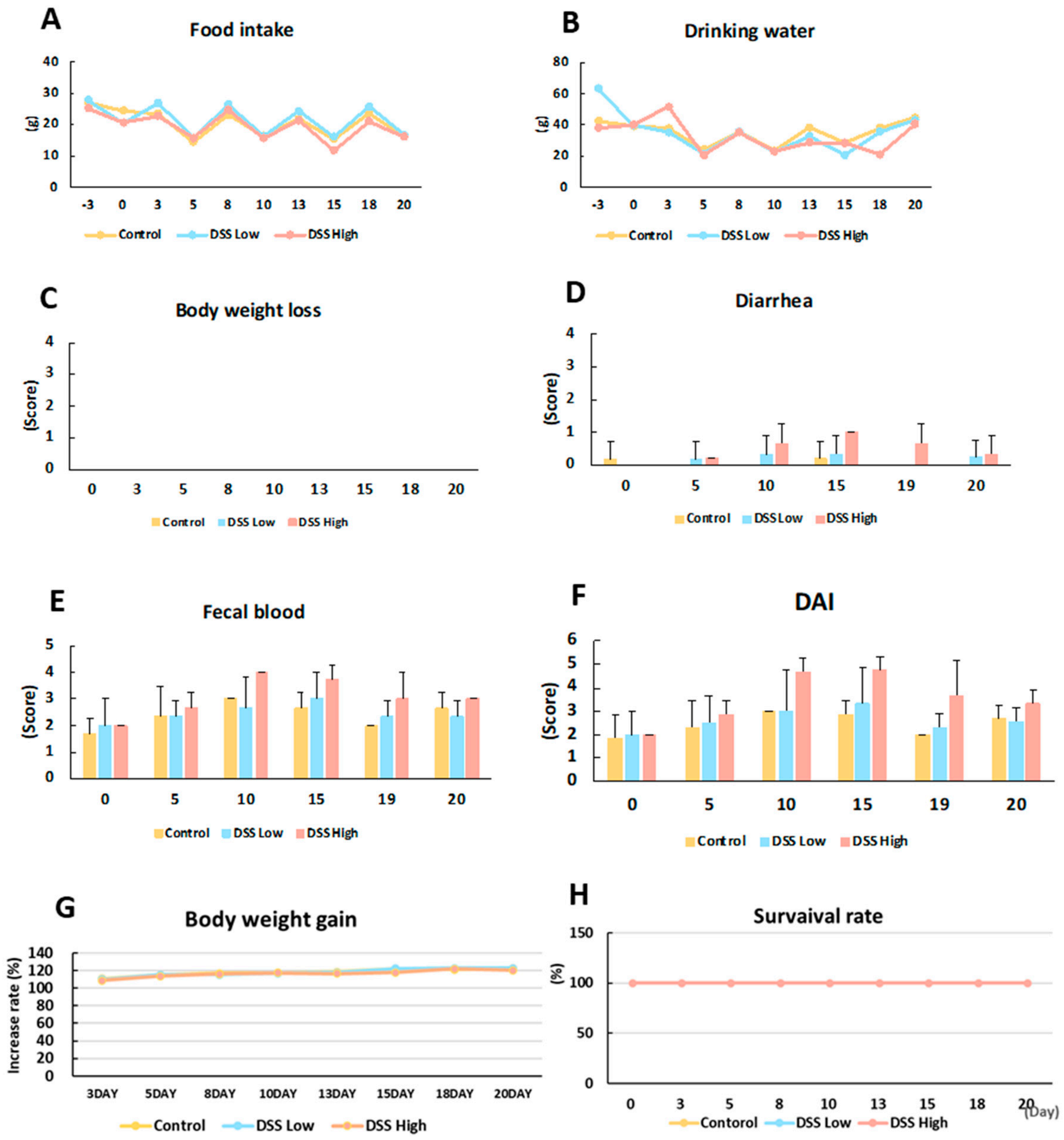

Supplemental Figure S3. Experiment II. Observation records from additional animal experiments for the purpose of obtaining genetic samples. The mice were treated as Experiment I (see Supplemental Figure S1). (A) Changes in food intake. (B) Changes in water intake. (C) Body weight loss (score). (D) Diarrhea (score). (E) Facial blood (score). (F) DAI was calculated from scores for weight loss, diarrhea, and facial blood (total score). The data represent the mean values for body weight loss scores, diarrhea scores, fecal blood scores, and DAI. (G) Body weight gain (%). The data represent the mean rate of increase. (H) Survival rate (%). *n*=6, control group (Control); *n*=6, 1.5; *n*=6, 3.0% DSS-treated group (DSS Low, DSS High).

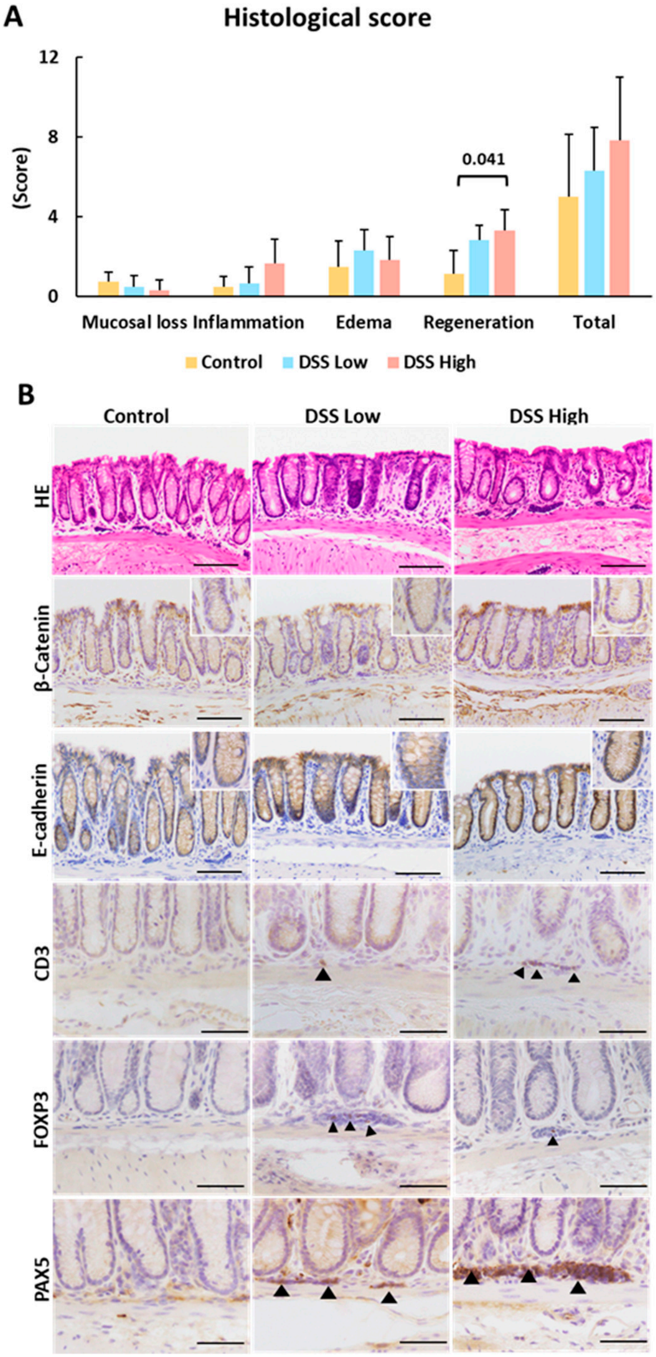

Supplementary Figure S4. Experiment I. Histological score and representative images of histopathology and immunohistochemistry. (A) Histological score. The data represent the mean values and standard deviations for the scores of mucosal loss, inflammation, edema, regeneration, and total score (see Supplemental Table S12). A significant difference between groups is shown as a p-value (Mann-Whitney U test with the Bonferroni correction for comparison between the three groups). (B) HE stain, and immunohistochemical staining of β-catenin, E-cadherin, CD3,

FOXP3, and PAX5 in Control, DSS Low, and DSS High. Clustered lymphocytes express CD3, FOXP3, and PAX5 (arrowheads) in the DSS-treated groups. Bar=200 μm (HE, β-catenin, and E-cadherin) and 50 μm (CD3, FOXP3, and PAX5). Control, the control group (*n*=4); DSS Low, the low dose DSS-treated group (*n*=6); and DSS High; the high dose DSS-treated group (*n*=6); FOXP3, forkhead box P; HE, hematoxylin and eosin; PAX5; Paired box protein 5.

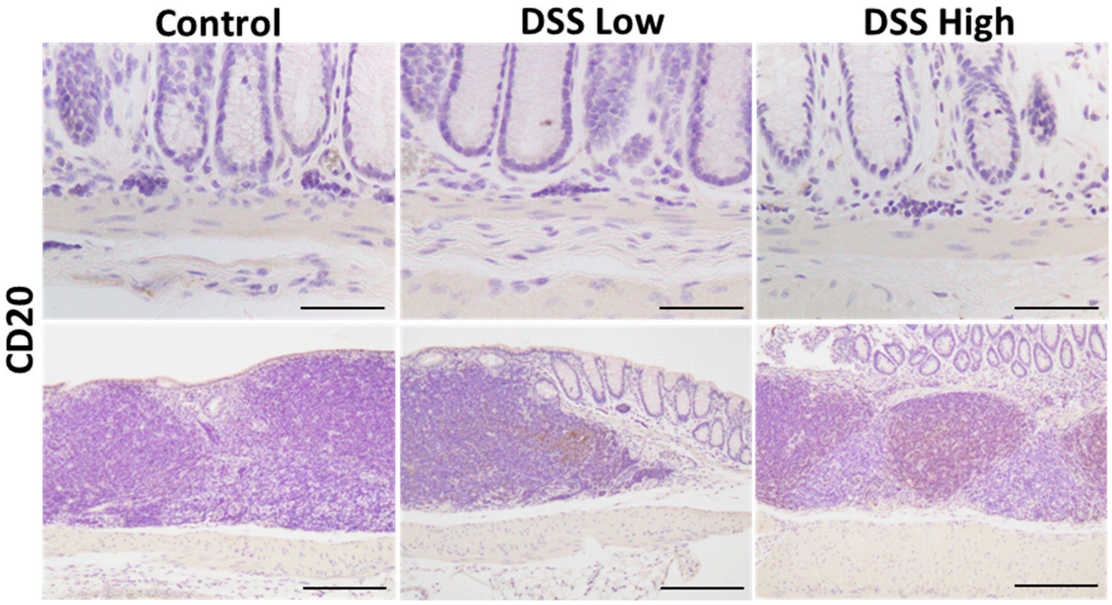

Supplemental Figure S5. Experiment I. Representative images of immunohistochemical staining of CD20 in Control, DSS Low, and DSS High. The upper row shows accumulated lymphocytes in lamina propria, and the lower row shows lymph follicles. Bar=50 µm (upper images) and 200 µm (lower images). Control, the control group (*n*=4); DSS Low, the low dose DSS-treated group (*n*=6); and DSS High; the high dose DSS-treated group (*n*=6).

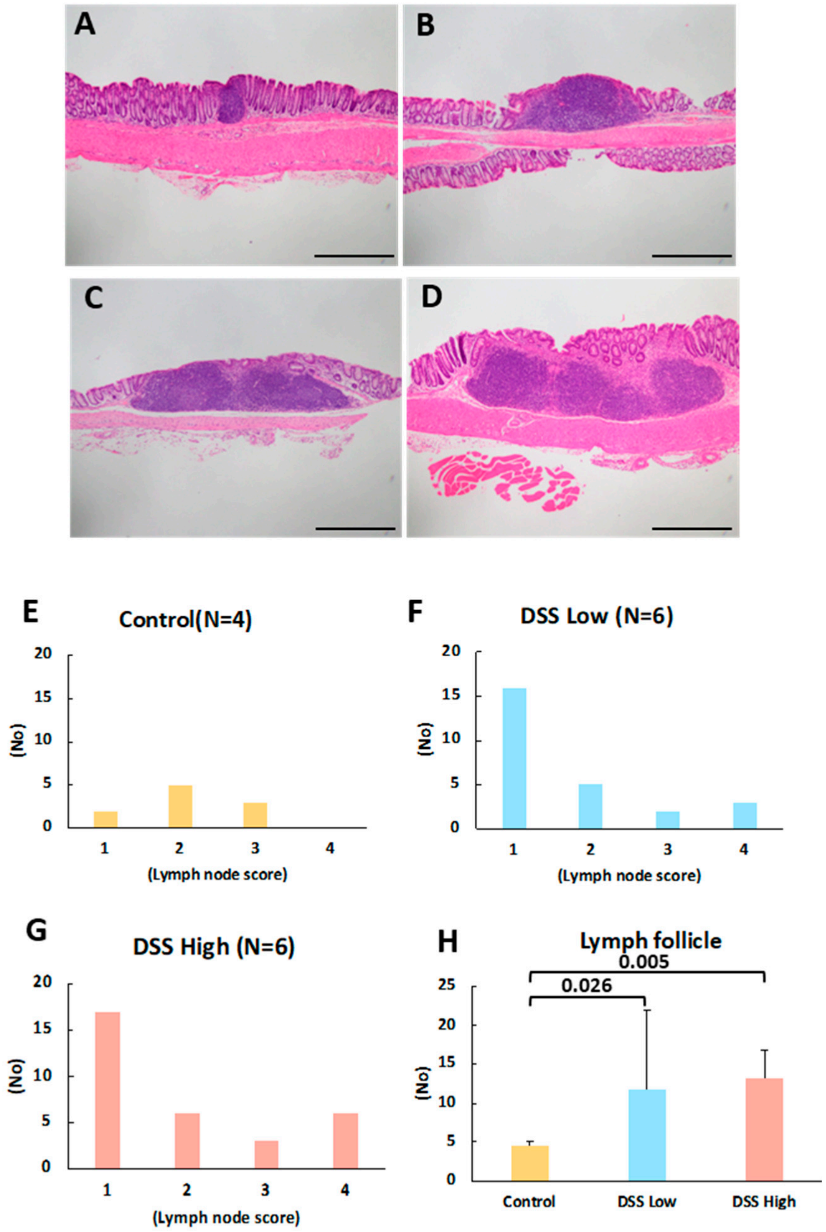

Supplemental Figure S6. Experiment I. Representative images of lymph follicles that serve as the basis for the scores in Supplemental Table S13. (A) Score 1: a single lymph follicle. (B) Score 2: a single large lymph follicle. (C) Score 3: chain of two lymph follicles. (D) Score 4: chains of three lymph follicles. Bar=500µm. (E-G) The number of lymph follicles is classified based on the scores in Supplemental Table S13. (H) The number of lymph follicles (total follicle count). The data represent the mean values and standard deviations. Significant differences between groups are shown as P values (Mann-Whitney U test with the Bonferroni correction for comparison among the three groups). Control, the control group (*n*=4); DSS Low, the low dose (1.5%) DSS-treated group (*n*=6); and DSS High; the high dose (3.0%) DSS-treated group (*n*=6).

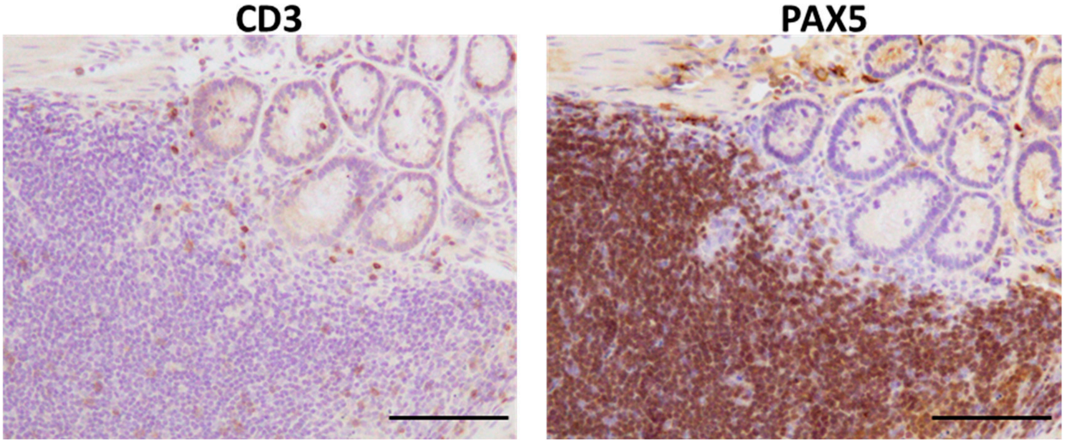

Supplemental Figure S7. Experiment I. Representative images of immunohistochemical staining of CD3 and PAX5 in the crypts close to lymphoid tissues. Bar＝100μm.

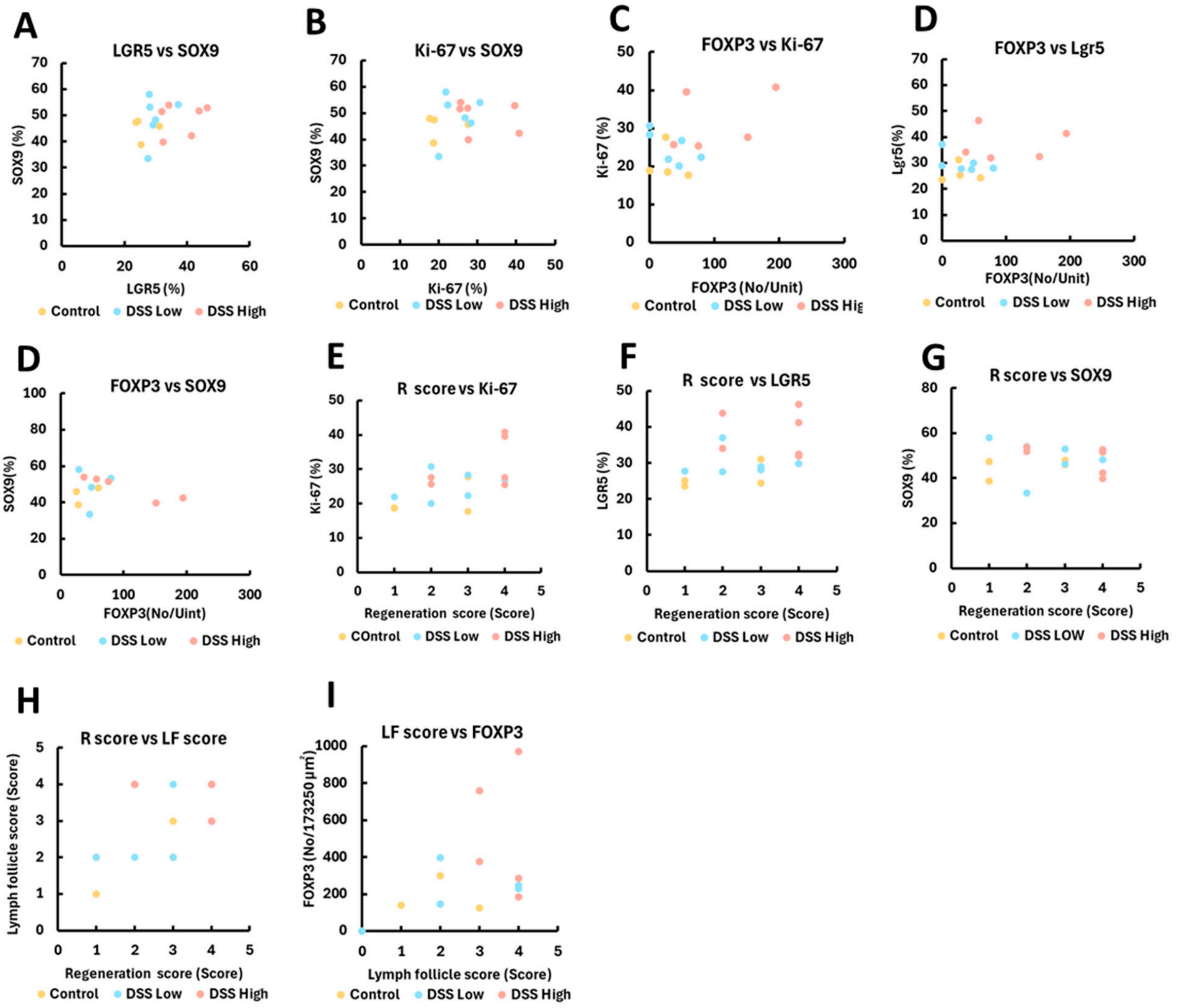

Supplemental Figure S8. Experiment I. Correlation plots of the respective positive cell rates for LGR5, SOX9, Ki-67, FOXP3, regenerative score, and lymphoid follicle score in Control, DSS Low, and DSS High. Control, the control group (*n*=4); DSS Low, the low dose (1.5%) DSS-treated group (*n*=6); and DSS High; the high dose (3.0%) DSS-treated group (*n*=6); FOXP3, forkhead box P; LF, Lymph follicle score; LGR5, leucine-rich repeat-containing G-protein coupled receptor 5; R score, Regeneration score; SOX9, sex-determining Region Y Box 9.

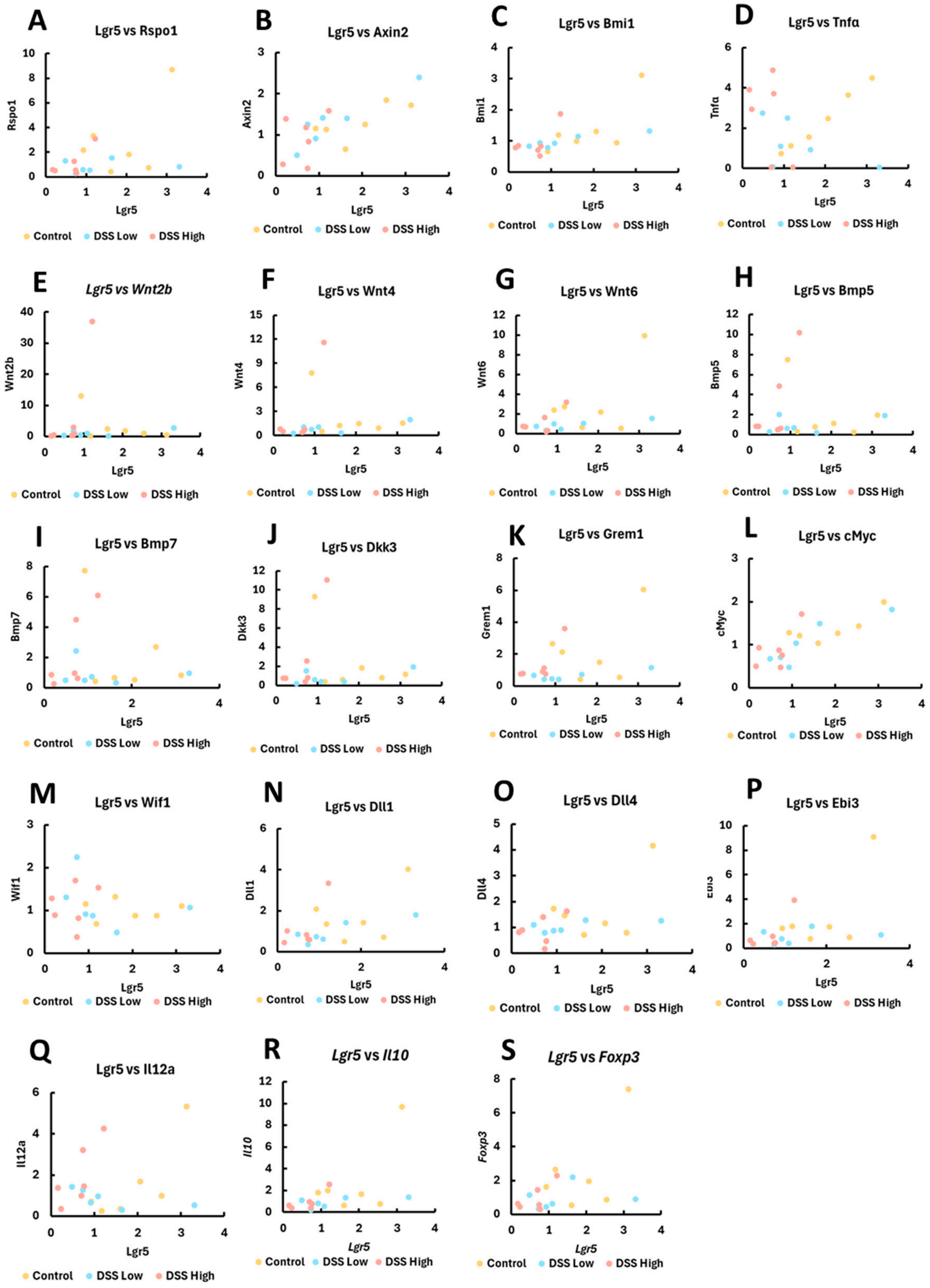

Supplemental Figure S9. Experiment II. Correlation diagram between the expression level of *Lgr5* gene and the expression level of other genes by RT-PCR (*n*=6 in each group).

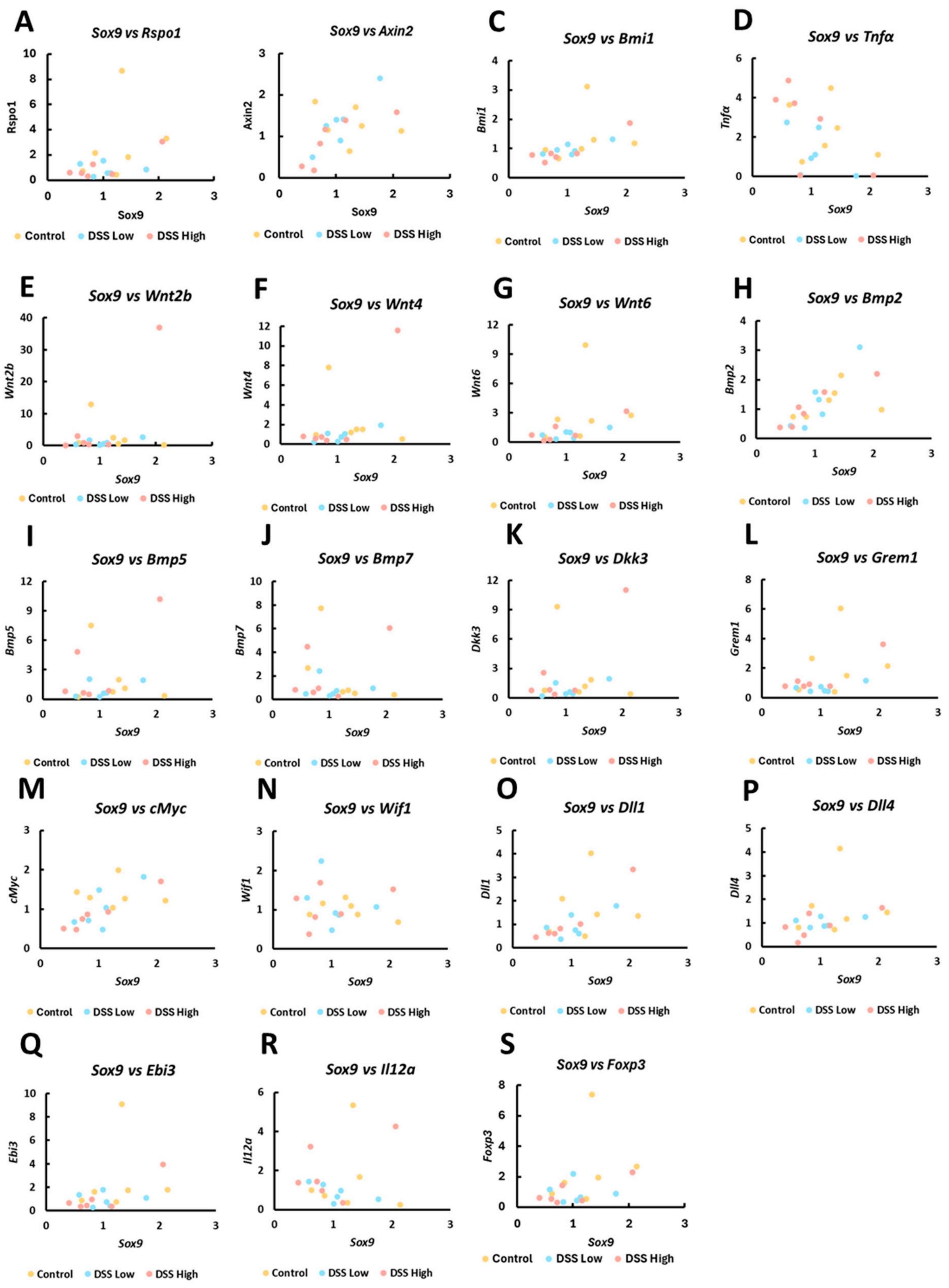

Supplemental Figure S10. Experiment II. Correlation diagram between the expression level of *Sox9* gene and the expression level of other genes by RT-PCR (*n*=6 in each group).

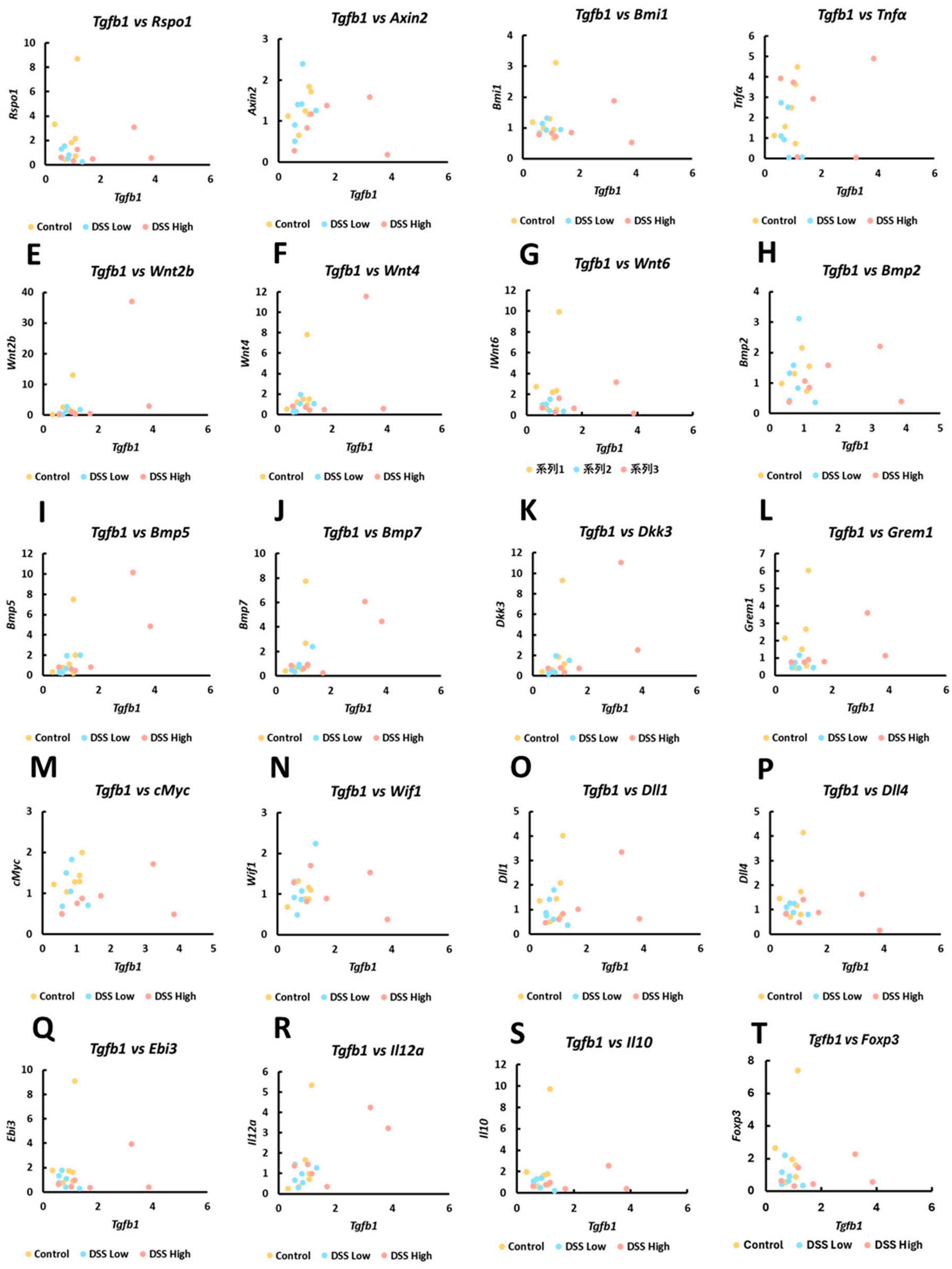

Supplemental Figure S11. Experiment II. Correlation diagram between the expression level of *Tgfb1* gene and the expression level of other genes by RT-PCR (*n*=6 in each group).

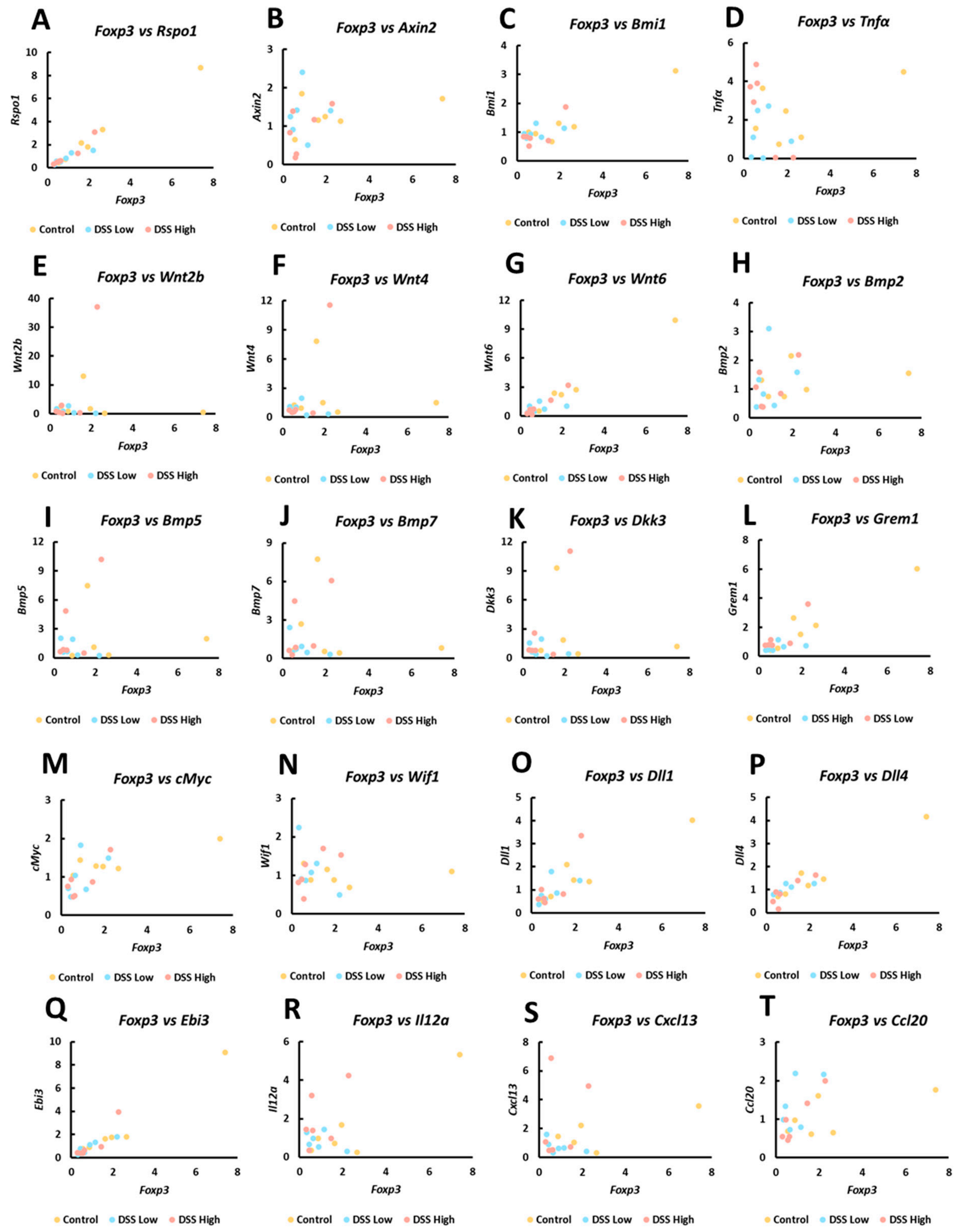

Supplemental Figure S12. Experiment II. Correlation diagram between the expression level of *Foxp3* gene and the expression level of other genes by RT-PCR (*n*=6 in each group).

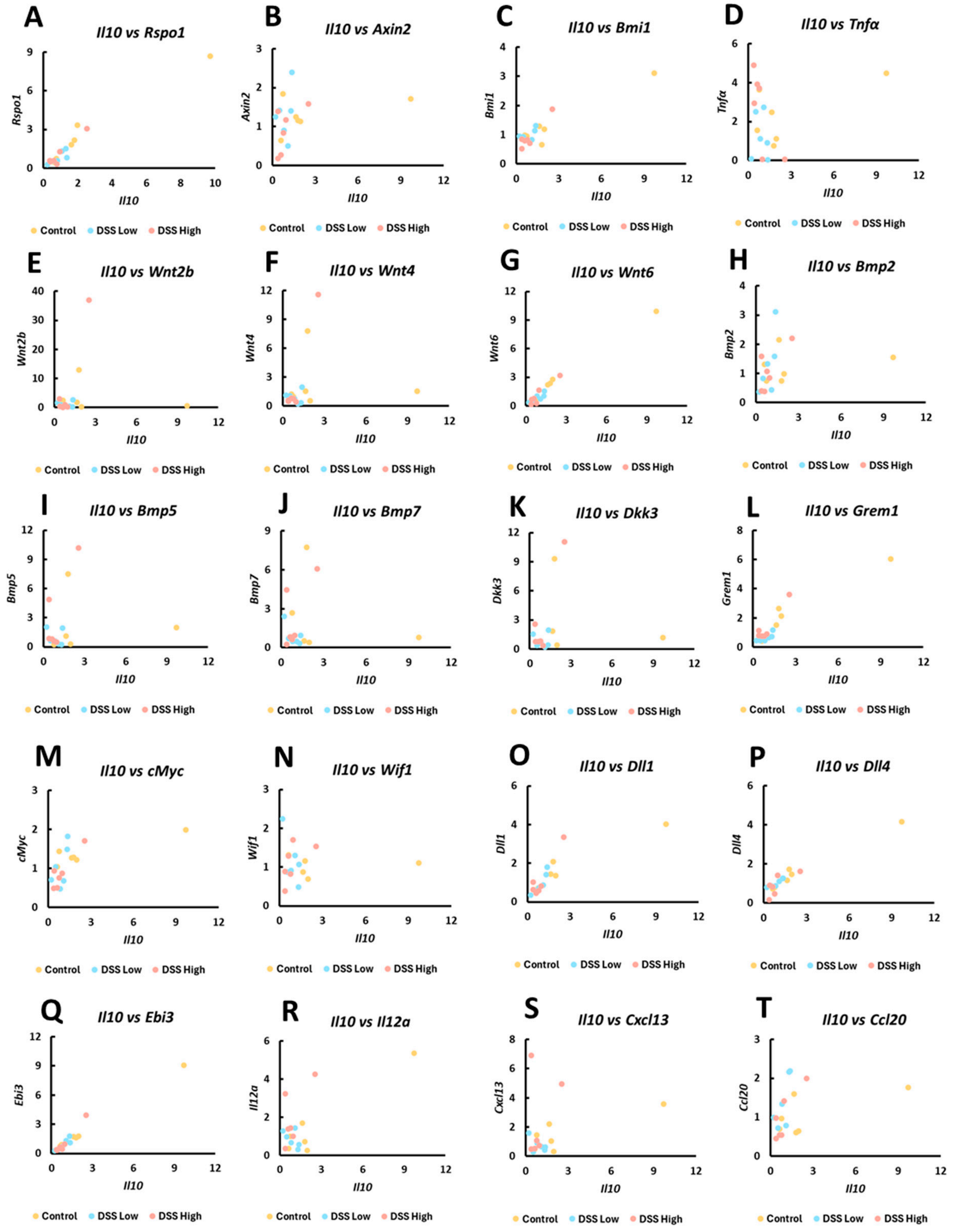

Supplemental Figure S13. Experiment II. Correlation diagram between the expression level of *Il10* gene and the expression level of other genes by RT-PCR (*n*=6 in each group).

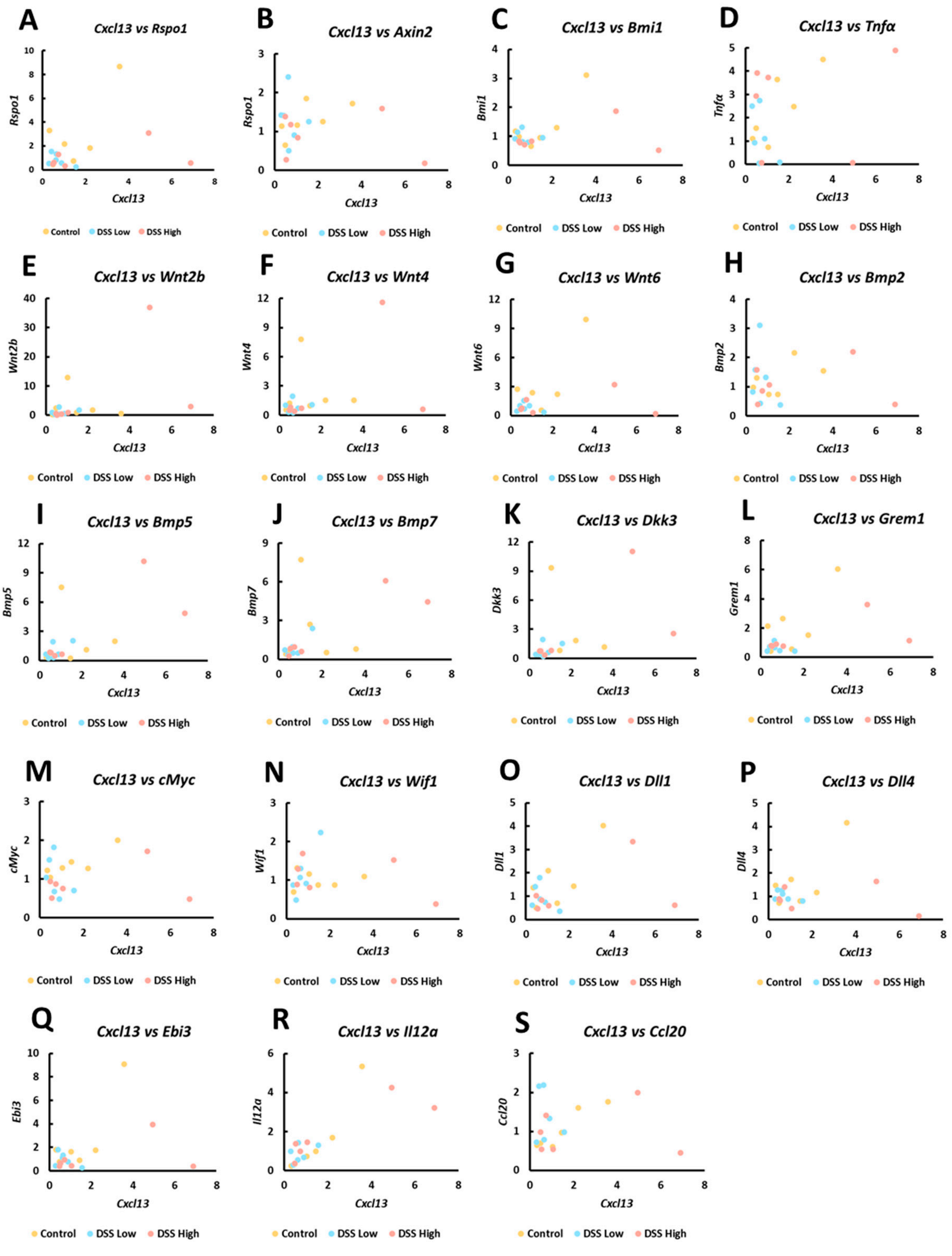

Supplemental Figure S14. Experiment II. Correlation diagram between the expression level of *Cxcl13* gene and the expression level of other genes by RT-PCR (*n*=6 in each group).

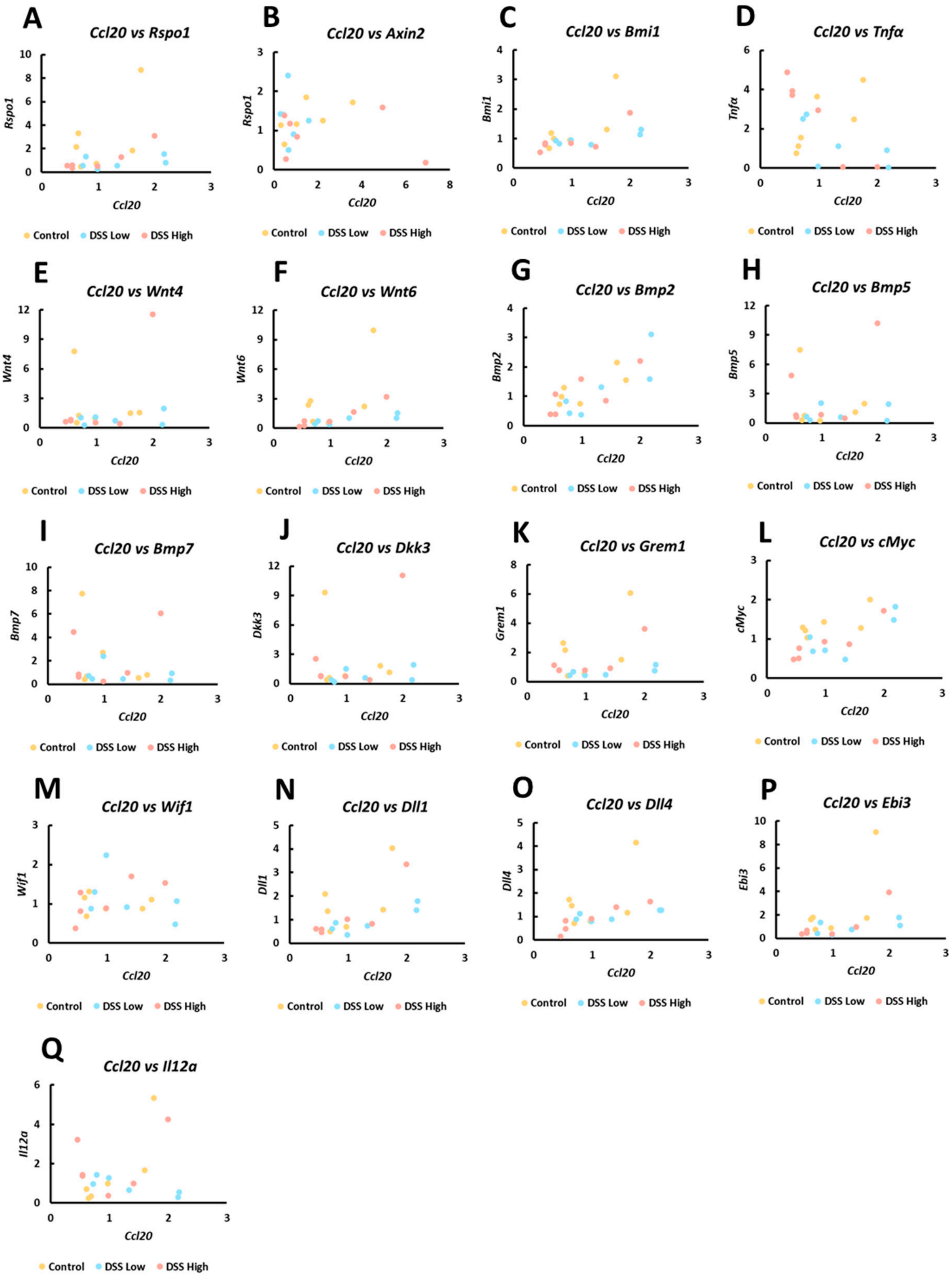

Supplemental Figure S15. Experiment II. Correlation diagram between the expression level of *Ccl20* gene and the expression level of other genes by RT-PCR (*n*=6 in each group).

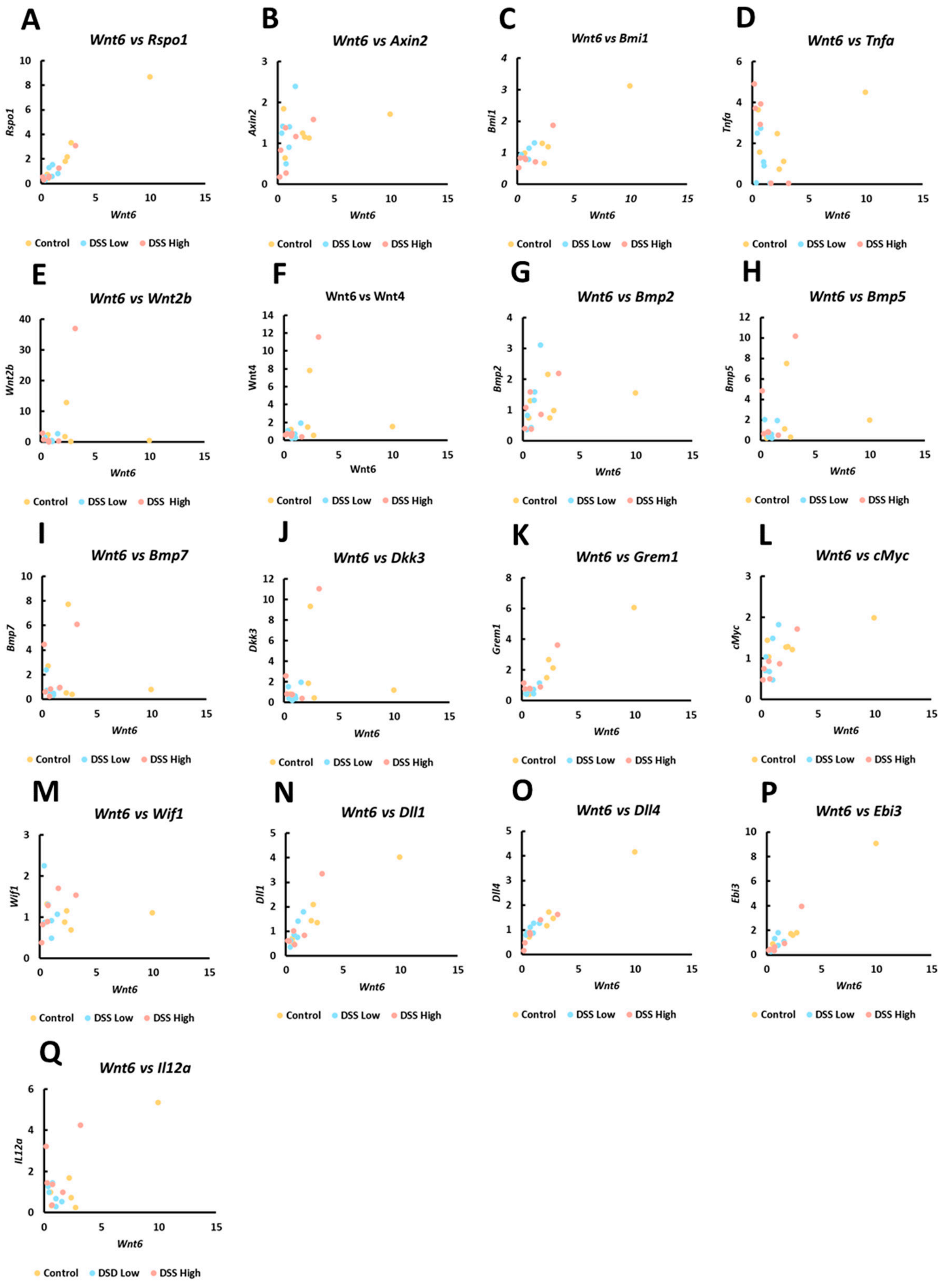

Supplemental Figure S16. Experiment II. Correlation diagram between the expression level of *Wnt6* gene and the expression level of other genes by RT-PCR (*n*=6 in each group).

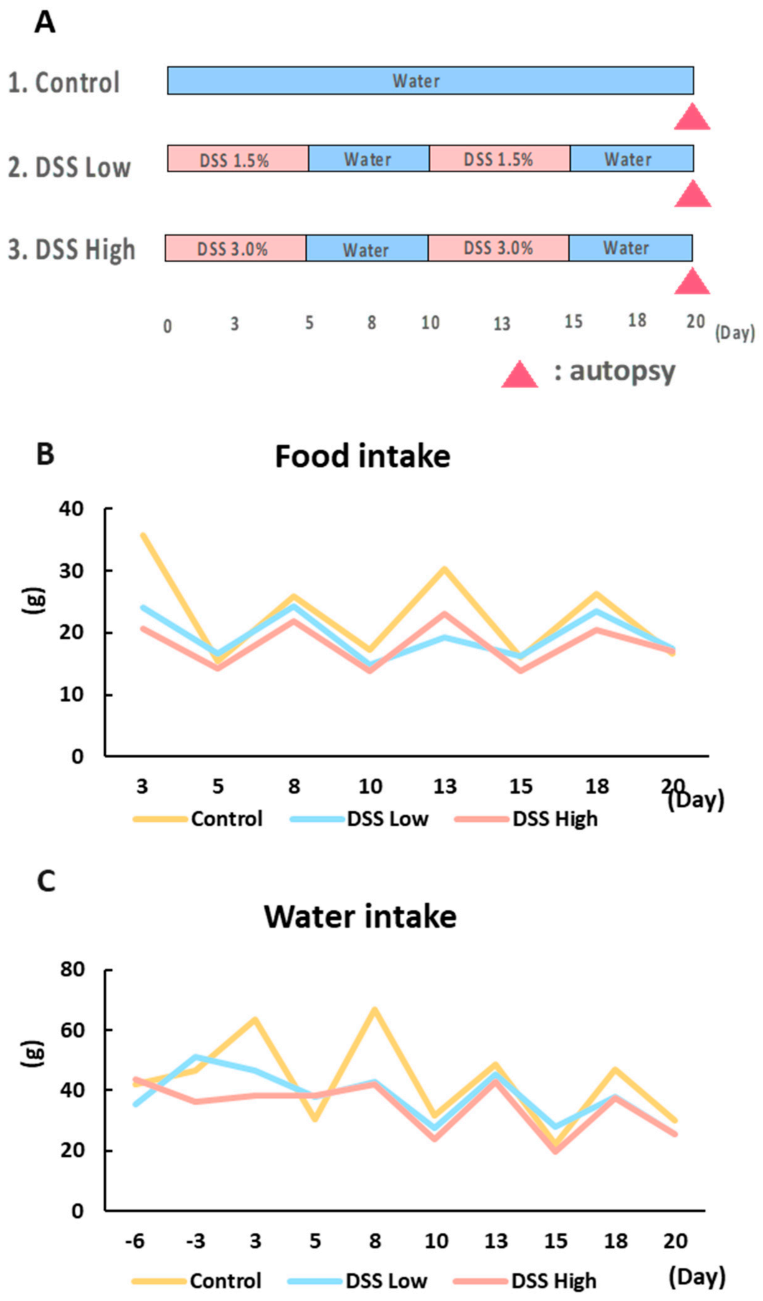

Supplemental Figure S17. (A) Study design for Experiment I/II. Treatments by group are shown. Animals were divided into three groups: control (*n*=4 for experiment I; *n*=6 for experiment II), low-dose DSS (*n*=6), and high-dose DSS (*n*=6) (Control, DSS Low, and DSS High, respectively). Two cycles of DSS treatment were given, with one cycle of administration of 1.5% (low-dose) and 3% (high-dose) DSS in water for 5 days, followed by a 5-day withdrawal. (B) Experiment I. Changes in food intake in Control, DSS Low, and DSS High. (C) Experiment I. Changes in water intake in each group.
